# The transcriptional coregulator PGC-1β controls mitochondrial function and anti-oxidant defence in skeletal muscles

**DOI:** 10.1038/ncomms10210

**Published:** 2015-12-17

**Authors:** Thanuja Gali Ramamoorthy, Gilles Laverny, Anna-Isabel Schlagowski, Joffrey Zoll, Nadia Messaddeq, Jean-Marc Bornert, Salvatore Panza, Arnaud Ferry, Bernard Geny, Daniel Metzger

**Affiliations:** 1 Institut de Génétique et de Biologie Moléculaire et Cellulaire, Centre National de la Recherche Scientifique UMR 7104, Institut National de la Santé et de la Recherche Médicale U964, Université de Strasbourg, 67400 Illkirch, France; 2 Institut de Physiologie EA 3072, Fédération de Médecine Translationnelle, Université de Strasbourg, Service de physiologie et d'Explorations Fonctionnelles, CHRU, 67000 Strasbourg, France; 3 Department of Pharmacy, Health and Nutritional Sciences, University of Calabria, Arcavacata di Rende, 87036 Cosenza, Italy; 4 Institut National de la Santé et de la Recherche Médicale U974, Centre National de la Recherche Scientifique UMR 7215, Université Pierre et Marie Curie-Paris 6 and Université Paris Descartes, 75006 Paris, France

## Abstract

The transcriptional coregulators PGC-1α and PGC-1β modulate the expression of numerous partially overlapping genes involved in mitochondrial biogenesis and energetic metabolism. The physiological role of PGC-1β is poorly understood in skeletal muscle, a tissue of high mitochondrial content to produce ATP levels required for sustained contractions. Here we determine the physiological role of PGC-1β in skeletal muscle using mice, in which PGC-1β is selectively ablated in skeletal myofibres at adulthood (PGC-1β^(i)skm−/−^ mice). We show that myofibre myosin heavy chain composition and mitochondrial number, muscle strength and glucose homeostasis are unaffected in PGC-1β^(i)skm−/−^ mice. However, decreased expression of genes controlling mitochondrial protein import, translational machinery and energy metabolism in PGC-1β^(i)skm−/−^ muscles leads to mitochondrial structural and functional abnormalities, impaired muscle oxidative capacity and reduced exercise performance. Moreover, enhanced free-radical leak and reduced expression of the mitochondrial anti-oxidant enzyme Sod2 increase muscle oxidative stress. PGC-1β is therefore instrumental for skeletal muscles to cope with high energetic demands.

## Abstract (web summary)

PGC-1 family transcription factors are important regulators of cellular energy metabolism. Here the authors use tissue-specific, inducible PGC-1β KO mice to show that PGC-1 family members are not functionally redundant and that PGC-1β is required to maintain mitochondrial function in skeletal muscle.

## Graphical abstract

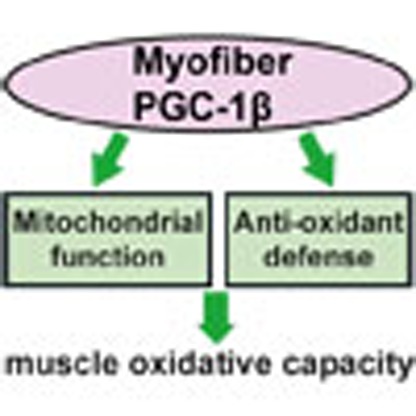

Peroxisome proliferator-activated receptor γ co-activator 1 (PGC-1) α and β are enriched in tissues with high oxidative capacity, such as brown adipose tissue, brain, heart and skeletal muscle^1^,^2^,^3^. Overexpression studies in mammalian cells in culture or in transgenic mice have shown that both PGC-1α and PGC-1β enhance the expression of a cascade of partially overlapping genes involved in mitochondrial biogenesis and respiratory function, through coactivation of numerous transcription factors^4^,^5^. PGC-1α controls adaptive thermogenesis in brown fat^3^, gluconeogenesis in liver^6^,^7^, oxidative metabolism in heart^8^ and protects from oxidative stress in various cell types^9^,^10^,^11^. PGC-1β regulates adaptive thermogenesis^12^, hepatic lipogenesis and lipoprotein secretion^13^, and cardiac contractile function following stress, such as pressure overload hypertrophy and β-adrenergic stimulation^14^,^15^. As PGC-1α- and PGC-1β-null mice only exhibit mild basal phenotypes, whereas mice bearing compound germ-line mutation of PGC-1α and PGC-1β die shortly after birth from heart failure, these coregulators exert redundant functions^16^.

Skeletal muscles are essential for postural maintenance, locomotion and respiration, and require high amounts of ATP during contractions. Mitochondria are the major site of oxidative metabolism to generate ATP, and a predominant source of reactive oxygen species (ROS), when electrons passing through the electron transport chain (ETC) leak out, leading to incomplete reduction of oxygen^17^,^18^,^19^,^20^,^21^,^22^. To limit oxidative stress, ROS generation is counterbalanced by the action of anti-oxidant enzymes^23^. Enhanced oxidative metabolism during intensive exercise generates high levels of ROS (refs 23, 24, 25), and induces an adaptive increase of ROS detoxifying enzyme levels and activity^26^,^27^,^28^,^29^.

Gain-of-function studies in skeletal muscles have shown that PGC-1α overexpression increases the proportions of type I and IIa myofibres, and activates oxidative metabolic genes^30^, whereas muscle specific PGC-1α ablation induced a fibre type shift from oxidative type I and IIa to glycolytic type IIx and IIb fibres^31^. PGC-1β overexpression in skeletal muscles during myofibre formation has been shown to promote oxidative type IIx fibres^32^. However, fibre type composition was not affected upon myofibre PGC-1β ablation in a PGC-1α -null background^33^, whereas the proportion of myosin heavy chain (MHC) I fibres was increased in PGC-1β-deficient myofibres of PGC-1α hypomorphic mice^34^. The slightly reduced muscle oxidative capacity in PGC-1β-deficient fibres was strongly enhanced by PGC-1α deficiency^34^. Surprisingly, muscle mitochondrial content was decreased in PGC-1β-deficient fibres in a PGC-1α hypomorphic, but not in null genetic background^33^,^34^. Due to these discrepancies in the literature, the physiological role of PGCs in skeletal muscles remains unclear.

To investigate the function of PGC-1β in mature skeletal myofibres, and avoid compensatory mechanisms that might occur during muscle fibre formation, we generated and characterized PGC-1β^(i)skm−/−^ mice, in which PGC-1β is selectively ablated in skeletal myofibres at adulthood. We show that fibre type composition and mitochondrial number are preserved in skeletal muscles of PGC-1β^(i)skm−/−^ mice. However, PGC-1β deficiency induced mitochondrial structural and functional abnormalities that decreased muscle oxidative capacity, leading to impaired exercise performance, but did not impact glucose homeostasis. Moreover, increased free-radical leak of the mitochondrial respiratory chain and reduced anti-oxidant defence in PGC-1β-deficient myofibres-induced oxidative stress. Thus, PGC-1 α and β are not fully redundant, and myofibre PGC-1β is required to maintain healthy populations of mitochondria in skeletal muscles.

## Results

### Myofibre composition is unaltered in PGC-1β^(i)skm−/−^ mice

**Figure 1:**
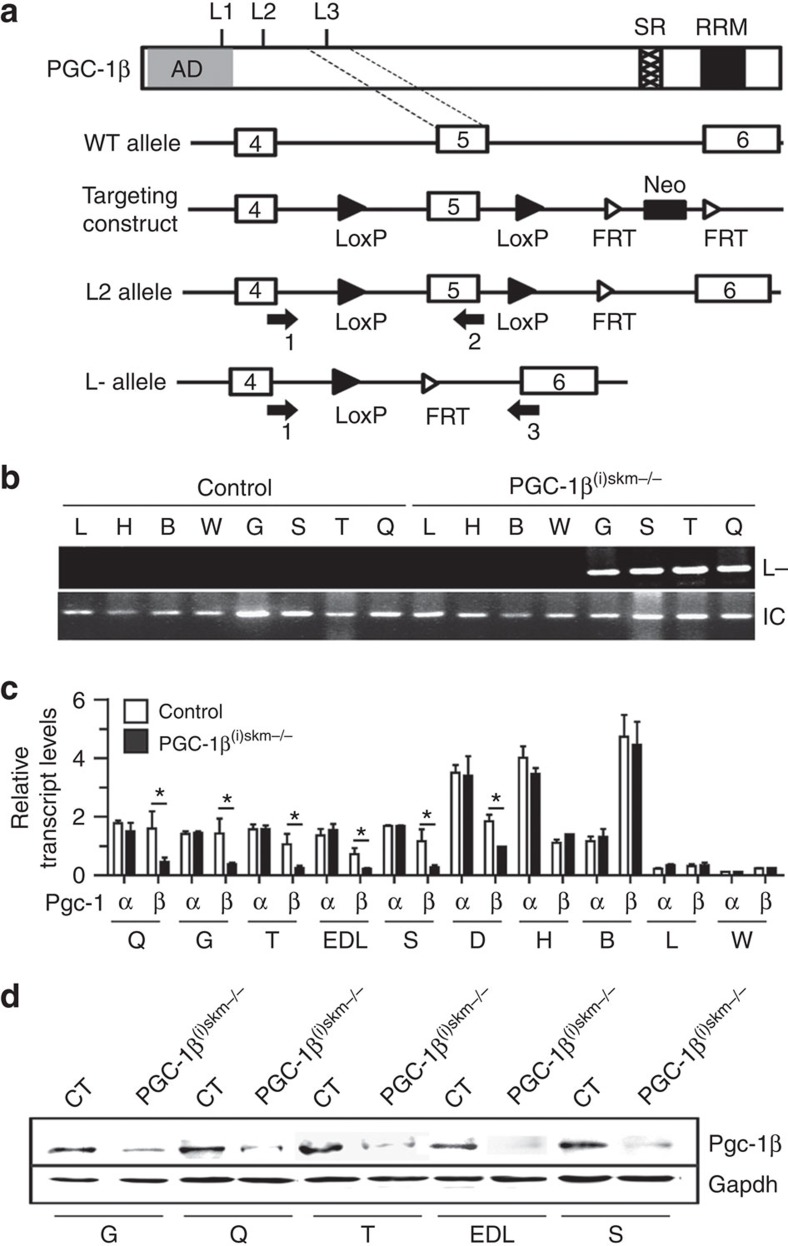
Characterization of Tam-induced Cre-ER^T2^-mediated PGC-1β ablation in skeletal myofibres of mice. (**a**) Schematic representation of the PGC-1β wild-type (WT) allele, the targeting construct, the floxed (L2) and the Cre-mediated exon 5 deleted (L-) *PGC-1β* alleles. L1, L2 and L3; LXXLL motifs. AD, activation domain; SR, serine arginine rich domain; RRM, RNA recognition motif; FRT, flippase recognition target. PCR primers 1–3 are indicated by arrows. Exons 4–6 are indicated by boxes, LoxP sites by closed arrow heads and FRT sites by open arrow heads. Neo, neomycin resistance gene. (**b**) Representative PCR detection of *PGC-1β* L- alleles in various organs of 11-week-old control and PGC-1β^(i)skm−/−^ mice. Gapdh is used as an internal control for DNA loading (*n*=10). (**c**) Relative PGC-1α and β transcript levels in various organs of 11-week-old control and PGC-1β^(i)skm−/−^ mice determined by RT-qPCR (*n*=10). (**d**) Representative western blot analysis of PGC-1β protein in skeletal muscles of 11-week-old control (CT) and PGC-1β^(i)skm−/−^ mice. Gapdh is used as the internal control (*n*=6). Data are represented as mean±s.e.m. *P<0.05, Student's *t*-test. B, brown adipose tissue; D, diaphragm; EDL, extensor digitorum longus; G, gastrocnemius; H, heart; L, Liver; Q, quadriceps; S, soleus; T, tibialis anterior; W, white adipose tissue.

To investigate the role of PGC-1β in skeletal myofibres of adult mice, PGC-1β^L2/L2^ mice bearing floxed *PGC-1β* L2 alleles (in which LoxP sites flank PGC-1β exon 5 encoding the third LXXLL nuclear receptor interaction domain) were intercrossed with human skeletal actin (HSA)-Cre-ER^T2(tg/0)^ mice, that express the Tamoxifen-dependent Cre-ER^T2^ recombinase selectively in myofibres^35^ (Fig. 1a). Male HSA-Cre-ER^T2(0/0)^/PGC-1β^L2/L2^ control mice and HSA-Cre-ER^T2(tg/0)^/PGC-1β^L2/L2^ somatic pre-mutant littermates were intraperitoneally injected at 7 weeks with 1 mg of Tamoxifen per day for 5 days to generate control and PGC-1β^(i)skm−/−^ mice, respectively. Conversion of *PGC-1β* L2 alleles into L- alleles occurred selectively in skeletal muscles of PGC-1β^(i)skm−/−^ mutant mice (Fig. 1b). PGC-1β transcript and protein levels were strongly decreased in various skeletal muscles of 8–26-week-old PGC-1β^(i)skm−/−^ mice compared with age-matched control mice, but not in other tissues such as liver, heart, brown and white adipose tissue (Fig. 1c,d, Supplementary Fig. 10a and data not shown). No compensatory increase of PGC-1α transcripts was observed in skeletal muscles of PGC-1β^(i)skm−/−^ mutant mice at 8–90 weeks of age (Fig. 1c and Supplementary Fig. 1a). Thus, induction of skeletal myofibre PGC-1β deficiency in adult mice does not alter PGC-1α expression.

The body weight of PGC-1β^(i)skm−/−^ and control mice fed a regular chow diet was similar over a period of 30 weeks (Supplementary Fig. 1b), and dual energy X-ray absortiometry scan analysis did not reveal any significant difference in body lean and fat contents between control and PGC-1β^(i)skm−/−^ mice at 26 weeks of age (Supplementary Fig. 1c). Food intake was slightly lower in mutants than in controls at 8–30 weeks of age, without reaching statistical significance (Supplementary Fig. 1d and data not shown). Moreover, skeletal muscle mass, myofibre structure and fibre cross-sectional area (CSA) distribution were not affected by PGC-1β ablation in myofibres (Supplementary Fig. 1e–i).

**Figure 2:**
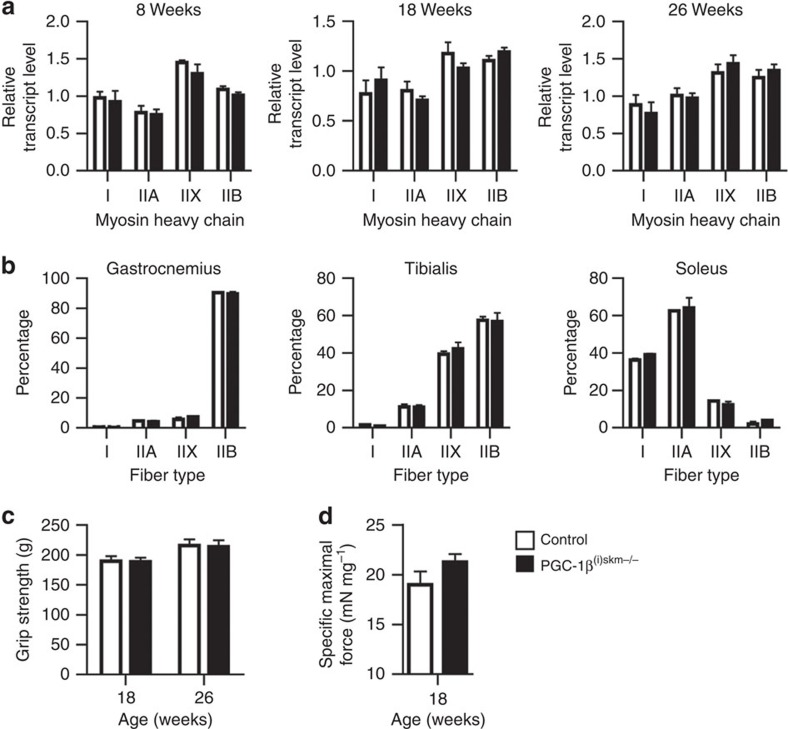
Characterization of PGC-1β^(i)skm−/−^ skeletal muscles. (**a**) Relative transcript levels of MHC type I, IIA, IIX and IIB in gastrocnemius muscle of 8, 18 and 26-week-old control and PGC-1β^(i)skm−/−^ mice determined by RT-qPCR (*n*=8). (**b**) Quantification of MHC I, IIA, IIX and IIB fibers by immunohistochemistry in gastrocnemius, tibialis anterior and soleus muscle of 26-week-old control and PGC-1β^(i)skm−/−^ mice (*n*=8). (**c**) Grip strength of control and PGC-1β^(i)skm−/−^ mice at 18 and 26 weeks of age (*n*=8). (**d**) *In situ* specific maximal force of tibialis anterior muscle from 18-week-old control and PGC-1β^(i)skm−/−^ mice (*n*=8). Data are represented as mean±s.e.m.

As overexpression of PGC-1β has been shown to drive the formation of type IIX fibres in skeletal muscle^32^, we determined MHC I, IIA, IIX and IIB expression at 8–26 weeks of age. No difference in their transcript levels was observed in gastrocnemius and tibialis anterior muscles between control and mutant mice (Fig. 2a and data not shown). Moreover, immunohistochemical quantification of MHC I-, IIA-, IIX- and IIB-positive fibres in gastrocnemius and tibialis anterior (fast twitch) and in soleus (slow twitch) at 26 weeks did not reveal any difference between control and PGC-1β^(i)skm−/−^ mice (Fig. 2b). Finally, grip strength and *in situ* maximal force of tibialis anterior muscle were similar in control and mutant mice (Fig. 2c,d).

Thus, myofibre PGC-1β does not control muscle fibre type composition and strength in adult mice.

### PGC-1β^(i)skm−/−^ muscles exhibit mitochondrial defects

**Figure 3:**
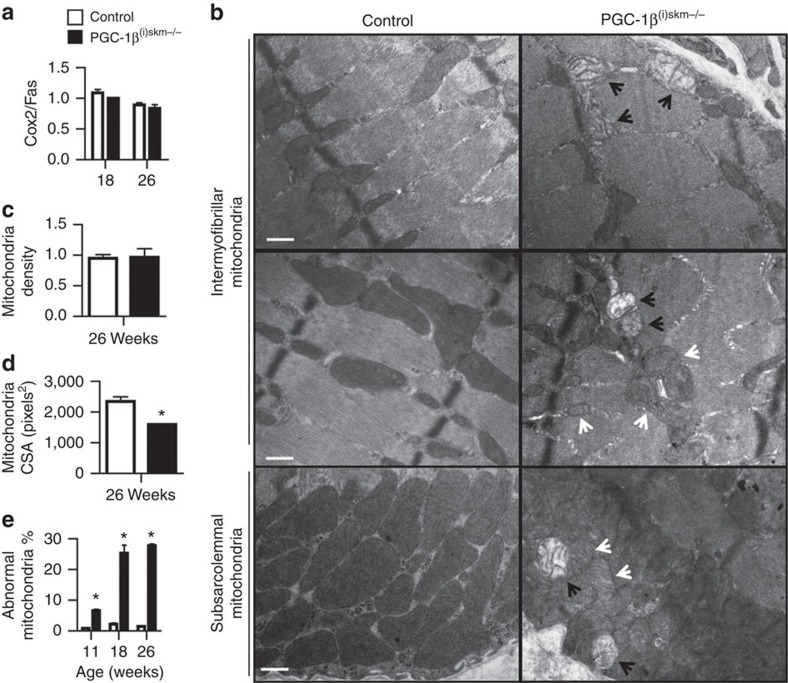
Characterization of mitochondria in PGC-1β^(i)skm−/−^ muscle. (**a**) Mitochondrial DNA content determined on gastrocnemius muscle DNA from 18 and 26-week-old control and PGC-1β^(i)skm−/−^ mice by quantitative PCR analysis using primers for *COX2* (mitochondrial gene) and *FAS* (nuclear gene) (*n*=18). (**b**) Representative electron micrographs of gastrocnemius muscle from 26-week-old control and PGC-1β^(i)skm−/−^ mice. Black arrows indicate mitochondria with disrupted cristae and white arrows indicate decreased cristae matrix density. Scale bars, 1 μm. (*n*=3). (**c**) Mitochondrial density per μm^2^ of fiber cross-section in gastrocnemius muscle of 26-week-old control and PGC-1β^(i)skm−/−^ mice determined on electron micrographs (*n*=3). (**d**) Mitochondria CSA in gastrocnemius muscle of 26-week-old control and PGC-1β^(i)skm−/−^ mice (*n*=5) evaluated on electron micrographs. (**e**) Quantification of abnormal mitochondria on electron micrographs of gastrocnemius muscle from 11, 18 and 26-week-old control and PGC-1β^(i)skm−/−^ mice (*n*=3). Data are represented as mean±s.e.m. *P<0.05, Student's *t*-test.

Muscle mitochondrial content, evaluated by real-time PCR with primers for cytochrome *c* oxidase subunit II (*Cox2* (mitochondrial gene)) and fatty-acid synthase (*Fas* (nuclear gene)), was similar in gastrocnemius, soleus, tibialis anterior and extensor digitorum longus of 18 and 26-week-old control and PGC-1β^(i)skm−/−^ mice (Fig. 3a and data not shown). Moreover, ultrastructural analysis did not reveal any difference in gastrocnemius mitochondrial density between control and mutant mice (Fig. 3b,c). However, the size of many intermyofibrillar and subsarcolemmal mitochondria was reduced in gastrocnemius muscle of 26-week-old PGC-1β^(i)skm−/−^ mice compared with control mice, and their structure was altered (Fig. 3b,d). Structural defects included cristae disruption (black arrows) and decreased cristae matrix density (white arrows) (Fig. 3b). At 11 weeks, ∼7% of the mitochondria were abnormal, a frequency that increased with time to reach ∼30% at 18–26 weeks (Fig. 3e).

**Figure 4:**
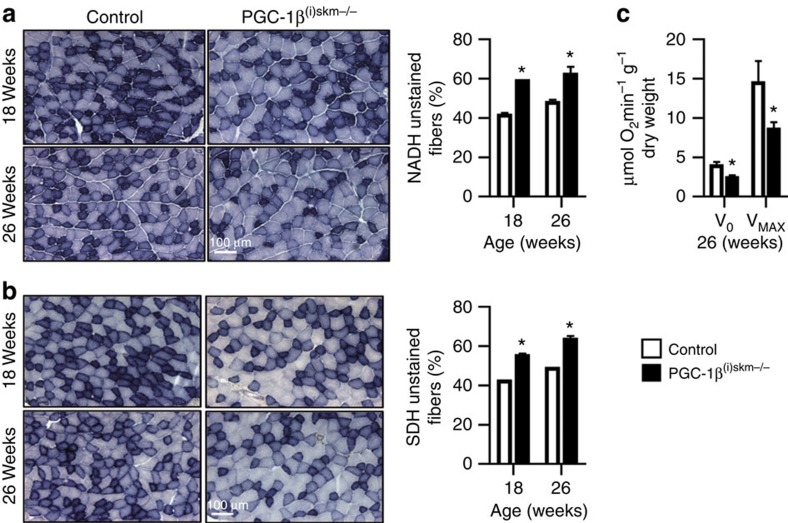
Oxidative capacity and mitochondrial respiration rate of PGC-1β^(i)skm−/−^ muscles. (**a**,**b**) Histological staining of tibialis anterior from 18 and 26-week-old control and PGC-1β^(i)skm−/−^ mice for NADH (**a**) and succinate (**b**) dehydrogenase activity (left panels), and quantification of unstained fibers (right panels; *n*=6). Scale bar, 100 μm. (**c**) Mitochondrial respiration rates determined on saponin-skinned quadriceps fibers of 26-week-old control and PGC-1β^(i)skm−/−^ mice in the absence (V_0_) and presence of ADP (V_MAX_). (*n*=9). Data are represented as mean±s.e.m. *P<0.05, Student's *t*-test.

Histochemical staining for the activity of NADH dehydrogenase and succinate dehydrogenase (SDH) (enzymes of complex I and II of the mitochondrial ETC, respectively), revealed a ∼15% increase of unstained fibres at both 18 and 26 weeks in tibialis anterior muscle of PGC-1β^(i)skm−/−^ mice as compared with control mice (Fig. 4a,b), indicating that oxidative capacity is decreased in myofibres of mutant mice. Moreover, mitochondrial respiration rates, determined in saponin-skinned quadriceps fibres from 26-week-old control and PGC-1β^(i)skm−/−^ mice, revealed that both basal (V_0_) and maximal (V_MAX_) oxygen consumption, measured in the absence and presence of ADP, respectively, were decreased by ∼40% in PGC-1β^(i)skm−/−^ mice compared with control mice (Fig. 4c). Taken together, these results demonstrate that myofibre PGC-1β is required to maintain a healthy population of mitochondria and basal muscle oxidative capacity.

### Many genes are downregulated in PGC-1β^(i)skm−/−^ muscles

**Figure 5:**
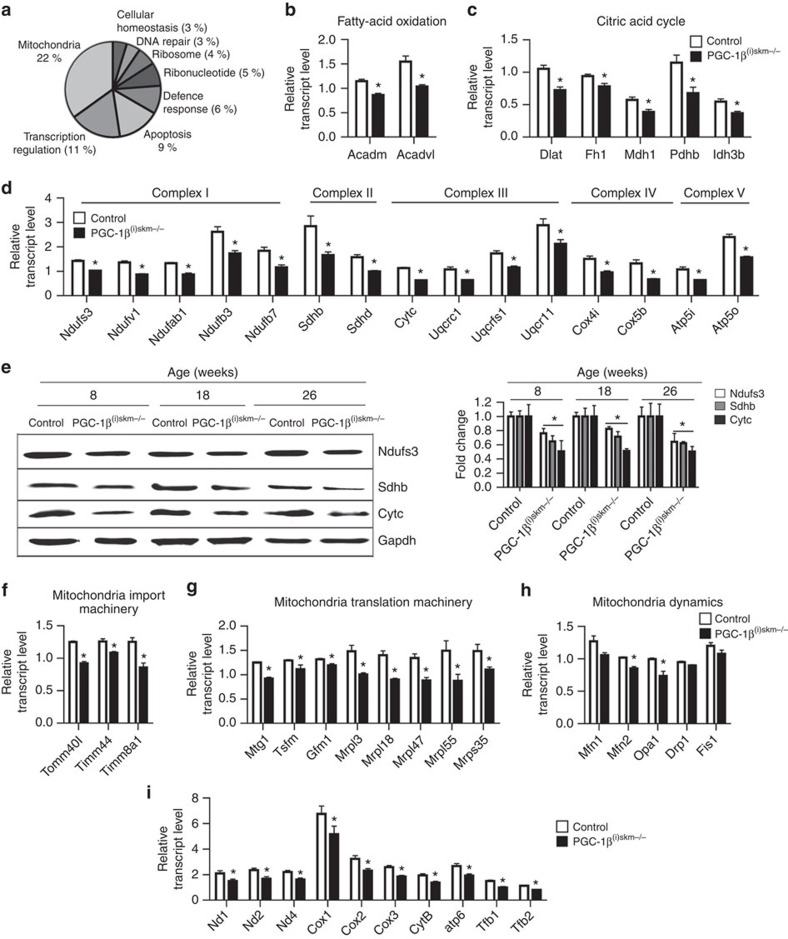
Impact of myofibre PGC-1β-deficiency on gene expression. (**a**) Pie chart of gene ontology annotation of genes downregulated by 1.2-fold in gastrocnemius of 8-week-old PGC-1β^(i)skm−/−^ mice compared with controls. (**b**–**d**) Relative transcript levels of genes encoding proteins involved in fatty-acid oxidation (*Acadm* and *Acadvl*; **b**) in citric acid cycle (*Dlat*, *Fh1*, *Mdh1*, *Pdhb* and *Idh3b*) (**c**) and Oxidative phosphorylation (OXPHOS; Complex I: *Ndufs3*, *Ndufv1*, *Ndufb3*, *Ndufb7*; Complex II: *Sdhb*, *Sdhd*; Complex III: *Cytc*, *Uqcrc1*, *Uqcrfs1*, *Uqcr11*; Complex IV: *Cox4i1*, *Cox5b*; and Complex V: *Atp5i*, *Atp5o*) (**d**) in gastrocnemius muscle of 8-week-old control and PGC-1β^(i)skm−/−^ mice, determined by RT-qPCR (*n*=8). (**e**) Representative western blot analysis of Ndufs3 (complex I), Sdhb (complex II) and Cytc (complex III) from quadriceps muscle of 8, 18 and 26-week-old control and PGC-1β^(i)skm−/−^ mice (left panel), and average fold-change (right panel; *n*=6). Gapdh is used as the internal control. (**f**) Relative transcript levels of *Tomm40l*, *Timm44* and *Timm8a1* in gastrocnemius muscle of 8-week-old control and PGC-1β^(i)skm−/−^ mice, determined by RT-qPCR (*n*=8). (**g**) Relative transcript levels of *Mtg1*, *Tsfm*, *Gfm1*, *Mrpl18*, *Mrpl47*, *Mrpl55* and *Mrps35* in gastrocnemius muscle of 8-week-old control and PGC-1β^(i)skm−/−^ mice, determined by RT-qPCR (*n*=8). (**h**) Relative transcript levels of *Mfn1*, *Mfn2*, *Opa1*, *Drp1* and *Fis1* in gastrocnemius muscle of 8-week-old control and PGC-1β^(i)skm−/−^ mice, determined by RT-qPCR (*n*=8). (**i**) Relative transcript levels of the mitochondrial encoded genes *Nd1*, *2* and *4*, *Cox1, 2* and *3*, *Cytb* and *Atp6*, and of the nuclear-encoded mitochondrial transcription factor *Tfb1* and *Tfb2*, in gastrocnemius muscle of 18-week-old control and PGC-1β^(i)skm−/−^ mice, determined by RT-qPCR (*n*=8). Data are represented as mean±s.e.m. *P<0.05, Student's *t*-test.

Transcriptional microarray analysis coupled with gene ontology annotation was conducted on gastrocnemius muscle of control and PGC-1β^(i)skm−/−^ mice 1 week after the last tamoxifen administration, to identify genes and signalling pathways that are deregulated upon PGC-1β ablation. Whereas, 346 nuclear genes were downregulated and 364 nuclear genes were upregulated by at least 1.2-fold in PGC-1β^(i)skm−/−^ mice, only 16 genes were downregulated and 4 upregulated by >1.5-fold. Among the main clusters of downregulated genes, genes encoding proteins involved in mitochondria integrity and functions (22%) and in transcriptional regulation (11%) were the most enriched (Fig. 5a). In contrast, no specific biological process, cellular components or molecular functions were enriched among the upregulated genes.

Quantitative PCR with reverse transcription (RT-PCR) analysis showed that the transcript levels of many nuclear genes controlling fatty-acid oxidation, the citric acid cycle and oxidative phosphorylation were decreased in gastrocnemius muscle of mutant mice, as well as in soleus, quadriceps and tibialis anterior muscles (Fig. 5b–d and Supplementary Fig. 2a–c). Moreover, protein levels of Ndufs3 (complex I of ETC) and of Sdhb (complex II) were reduced by∼20% in quadriceps muscle of mutant mice at 8 and 18 weeks, and by∼50% at 26 weeks, whereas those of Cytc (complex III) were decreased by ∼50% at 8–26 weeks (Fig. 5e and Supplementary Fig. 10b). In addition, the transcript levels of genes encoding proteins involved in mitochondrial protein import, such as Tomm40l, Timm44 and Timm8a1 and in mitochondrial translational machinery, such as Mtg1, Tsfm, Gfm1, Mrpl3, Mrpl18, Mrpl47, Mrpl55 and Mrps35, were reduced in skeletal muscles of PGC-1β^(i)skm−/−^ mice (Fig. 5f,g). Moreover, the transcript levels of mitofusin 2 (*Mfn2*) and optic atrophy 1 (*Opa1*), which encode two proteins involved in mitochondrial fusion, were decreased in skeletal muscles of mutant mice at 8, 18 and 26 weeks (Fig. 5h and Supplementary Fig. 3a,b). The transcript levels of *Mfn1* encoding another protein in mitochondrial fusion was also decreased at 8, 18 and 26 weeks, even though it reached statistical significance only at 18 weeks. In contrast, those of dynamin-related protein (*Drp1*) and Fission1 (*Fis1*) encoding two proteins controlling mitochondrial fission, were similar in control and mutant mice at 8–18 weeks of age, but were reduced at 26 weeks, suggesting that PGC-1β does not directly control their expression (Fig. 5h and Supplementary Fig. 3a,b). Note that our microarray data did not reveal any difference in the transcript levels of phosphatidylglycerophosphate synthase 1 (*Pgs*) and of protein tyrosine phosphatase localized to mitochondrion 1 (*Ptpmt*) or of taffazin (*Taz*) and lysocardiolipin acyltransferase 1 (*Lclat1*), controlling, respectively, the synthesis and remodelling of cardiolipin, a protein involved in mitochondrial bioenergetics processes and in mitochondrial membrane stability and dynamics^36^,^37^. Moreover, the transcript levels of *Pgs*, *Ptpmt*, *Taz* and *Lclat1*, analysed by quantitative RT-PCR, were similar in gastrocnemius muscles from control and mutant mice at 8, 18 and 26 weeks (Supplementary Fig. 3c–e). In addition, the transcript levels of transcription factors involved in mitochondrial biogenesis (for example, nuclear respiratory factor 1 (*NRF-1*), GA-binding protein transcription factor, alpha subunit (*Gabpa*, also referred as *NRF-2*), estrogen-related receptors (*ERRs*) and mitochondrial transcription factor *Tfam*) were similar in gastrocnemius muscle of control and PGC-1β^(i)skm−/−^ mice at 8, 18 and 26 weeks (Supplementary Fig. 4a–c).

Even though the transcript levels of mitochondria encoded proteins were similar in gastrocnemius muscle from control and mutant mice at 8 weeks (Supplementary Fig. 4d), they were reduced by 1.3–1.7-fold in gastrocnemius muscle from mutant mice at 18 and 26 weeks (Fig. 5i and Supplementary Fig. 4e). Interestingly, those of *Tfb1* and 2, two nuclear-encoded mitochondrial transcription factors, were decreased by 1.3-fold at 18 weeks and by 1.4 and 1.5-fold at 26 weeks, respectively (Fig. 5i and Supplementary Fig. 4e). In agreement with these results, the number of abnormal mitochondria was much higher at 18 and 26 weeks than at earlier time (Fig. 3e).

**Figure 6:**
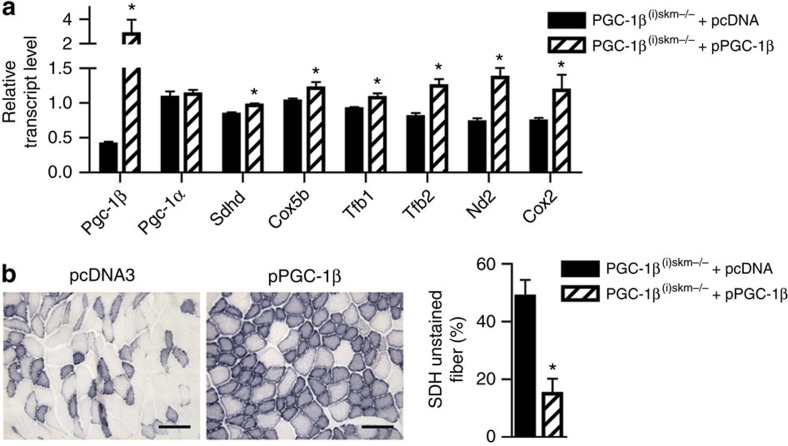
Rescue of mitochondrial defects in skeletal muscles of PGC-1β^(i)skm−/−^ mice. (**a**) Relative transcript levels, determined by RT-qPCR, of *PGC-1β* and *α*, *Sdhd*, *Cox5b*, *Tfb1*, *Tfb2*, *Nd2* and *Cox2* in gastrocnemius muscle of 26-week-old PGC-1β^(i)skm−/−^ mice, 7 days after electroporation with an empty vector (pcDNA3) or a plasmid encoding PGC-1β (pPGC-1β; *n*=4). (**b**) Histological staining of tibialis anterior from 18-week-old PGC-1β^(i)skm−/−^ mice, 7 days after electroporation with an empty vector (pcDNA3, left panel) or a plasmid encoding PGC-1β (right panel), for succinate dehydrogenase activity, and quantification of unstained fibers (*n*=4). Scale bar, 100 μm. Data are represented as mean±s.e.m. *P<0.05, Student's *t*-test.

To demonstrate that gene dysregulation in mutant mice is indeed induced by myofibre PGC-1β deficiency, we performed rescue experiments in PGC-1β^(i)skm−/−^ mice. PGC-1β transcript levels were increased by sevenfold in tibialis anterior muscle of 26-week-old PGC-1β^(i)skm−/−^ mice, 7 days after its electroporation with a PGC-1β expression vector, whereas those of nuclear (for example, *Sdhd*, *Cox5b*, *Tfb1* and *Tfb2*) and mitochondria (for example, *Nd2* and *Cox2*) encoded genes were 1.3–2-fold increased (Fig. 6a). In contrast, those of PGC-1α were unaffected (Fig. 6a). In addition, the number of unstained SDH fibres was 30% reduced by re-expressing PGC-1β in tibialis anterior muscle of PGC-1β^(i)skm−/−^ mice (Fig. 6b).

Taken together, these results show that myofibre PGC-1β enhances the expression of nuclear genes encoding proteins involved in mitochondrial gene expression and protein synthesis, import machinery and dynamics, and myofibre energy processes.

### PGC-1β deficiency does not impact glucose homeostasis

Since decreased expression of PGC-1α and β, and of other genes involved in oxidative phosphorylation, as well as mitochondrial dysfunction, have been proposed to play a causal role in the pathogenesis of type 2 diabetes^38^,^39^, we analysed glucose homeostasis in PGC-1β^(i)skm−/−^ mice. No difference in fasting glucose and insulin levels were observed between control and PGC-1β^(i)skm−/−^ mice both under regular diet and high-fat diet (HFD)-fed conditions (Supplementary Fig. 5a–d). Moreover, glucose tolerance and insulin sensitivity were similar between control and mutant mice, both under regular diet and HFD-fed conditions (Supplementary Fig. 5e–h). Note that the body weight, as well as muscle weight and histology of PGC-1β^(i)skm−/−^ mice were similar to those of control mice when fed a HFD (Supplementary Fig. 5i–k). Thus, even though mitochondrial functions are impaired in PGC-1β^(i)skm−/−^ myofibres, body weight and glucose homeostasis are not affected.

### Myofibre oxidative stress is enhanced in PGC-1β^(i)skm−/−^ mice

**Figure 7:**
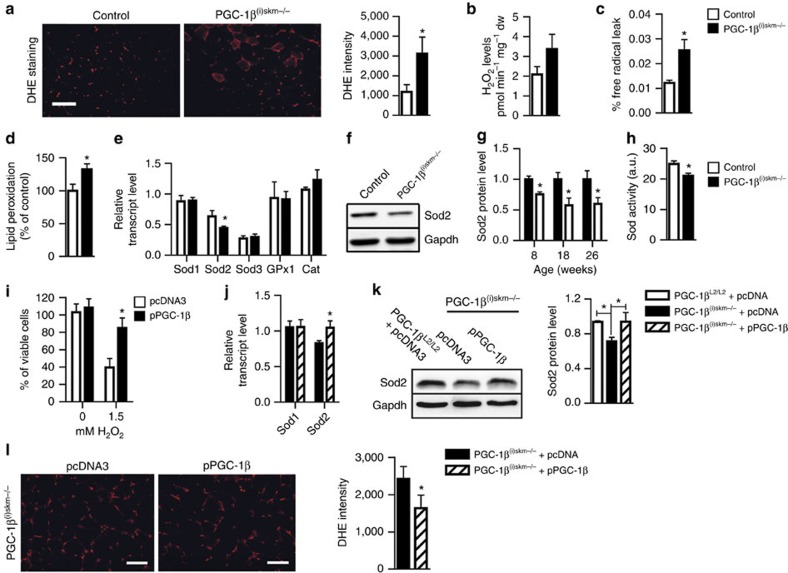
Oxidative stress in muscles of sedentary PGC-1β^(i)skm−/−^ mice. (**a**) DHE staining of gastrocnemius muscle sections from 26-week-old sedentary control and PGC-1β^(i)skm−/−^ mice (left panel), and staining intensity quantification (right panel; *n*=9). (**b**) H_2_O_2_ levels determined by Amplex Red, in gastrocnemius muscle of 26-week-old sedentary control and PGC-1β^(i)skm−/−^ mice (*n*=9). (**c**) Free-radical leak determined on saponin-skinned gastrocnemius fibers of 26-week-old sedentary control and PGC-1β^(i)skm−/−^ mice (*n*=9). (**d**) Lipid peroxidation, determined by Thiobarbituric Acid Reactive Substances Assay, in gastrocnemius muscle of 26-week-old sedentary control and PGC-1β^(i)skm−/−^ mice (*n*=9). (**e**) Relative transcript levels, determined by RT-qPCR, of *Sod 1*, *2*, *3*, *Gpx1* and catalase (Cat) in gastrocnemius muscle of 26-week-old sedentary control and PGC-1β^(i)skm−/−^ mice (*n*=8). (**f**) Representative western blot analysis of Sod2 from quadriceps muscle of 18-week-old control and PGC-1β^(i)skm−/−^ mice. Gapdh is used as the internal control. (**g**) Relative Sod2 protein levels in quadriceps muscle of 8, 18 and 26-week-old PGC-1β^(i)skm−/−^ mice and control mice. Gapdh is used as the internal control (*n*=6). (**h**) Sod activity, determined by a colorimetric reaction, in tibialis anterior muscle of 18-week-old PGC-1β^(i)skm−/−^ and control mice (*n*=4). (**i**) Viability of C_2_C_12_ myotubes transfected with an empty vector (pcDNA3) or a plasmid encoding PGC-1β (pPGC-1β) and cultured 6 h in absence or in presence of 1.5 mM H_2_O_2_, determined by trypan blue exclusion (*n*=4). (**j**) Relative transcript levels, determined by RT-qPCR, of *Sod1* and *2* in gastrocnemius muscle of 26-week-old PGC-1β^(i)skm−/−^ mice, 7 days after electroporation with pcDNA3 or pPGC-1β (*n*=4). (**k**) Representative western blot analysis of Sod2 in tibialis anterior muscle of 26-week-old control mice electroporated with pcDNA3, and PGC-1β^(i)skm−/−^ mice electroporated with pcDNA3 or pPGC-1β (left) and average fold-change (right). Gapdh is used as the internal control. (*n*=4). (**l**) DHE staining of gastrocnemius section from 18-week-old PGC-1β^(i)skm−/−^ mice electroporated with pcDNA3 or pPGC-1β (left panel), and staining intensity quantification (right panel; *n*=4). Scale bars, 100 μm. Data are represented as mean±s.e.m. *P<0.05, Student's *t*-test.

To investigate whether muscle mitochondrial structural and functional defects in PGC-1β^(i)skm−/−^ mice were associated with increased oxidative stress, intracellular ROS was quantified by dihydroethidium (DHE) staining. DHE reacts with superoxide anions to form a red fluorescent product (ethidium) which intercalates with DNA (ref. 40). A 2- and 1.8-fold increase in DHE staining intensity was observed in gastrocnemius and soleus of mutant mice at 26 weeks of age, respectively (Fig. 7a and Supplementary Fig. 6a). Moreover, hydrogen peroxide (H_2_O_2_) levels, quantified using the horseradish peroxidase (HRP; Invitrogen)/Amplex red assay, were slightly increased in gastrocnemius and soleus muscles of mutant mice as compared with control mice, even though it did not reach statistical significance (Fig. 7b and Supplementary Fig. 6b). Increased H_2_O_2_ levels result from higher production of superoxide by leaking electrons of the respiratory chain that react with oxygen^22^ and/or enhanced dismutation. Interestingly, the free-radical leak, corresponding to the percentage of electrons that react with oxygen to produce superoxide^17^, evaluated by normalizing H_2_O_2_ production to O_2_ consumption, was increased in gastrocnemius muscle of PGC-1β^(i)skm−/−^ mice (Fig. 7c). Increased ROS levels in skeletal muscles of PGC-1β^(i)skm−/−^ mice had a functional impact, as lipid peroxidation was 30% higher in tibialis anterior muscle of mutant mice than of control mice at 26 weeks (Fig. 7d). To characterize anti-oxidant defences in skeletal muscles of PGC-1β^(i)skm−/−^ mice, the transcript levels of superoxide dismutases, glutathione peroxidase 1 (*Gpx1*) and catalase were determined. Whereas those of the copper/zinc superoxide dismutase (*Sod1*), extracellular Sod (*Sod3*), Gpx1 and catalase were similar in control and PGC-1β^(i)skm−/−^ mice at 8–26 weeks of age, those of manganese superoxide dismutase (*Sod2*) were decreased in PGC-1β^(i)skm−/−^ mice (Fig. 7e and data not shown). In agreement with these results, Sod2 protein levels were ∼25–45% lower at 8–26 weeks of age, and Sod activity was reduced by 16% in tibialis anterior muscle of mutant mice at 18 weeks (Fig. 7f–h and Supplementary Fig. 10c). Thus, increased free-radical leak in mitochondria of skeletal muscles from PGC-1β^(i)skm−/−^ mice and decreased anti-oxidant defence enhanced muscle oxidative stress.

To demonstrate that PGC-1β protect myofibres from oxidative stress, C_2_C_12_ cells were transfected with a PGC-1β expression vector or an empty vector, differentiated for 3 days and treated with 1.5 mM H_2_O_2_ for 6 h. Whereas >60% of the cells transfected with an empty-vector died after such a treatment, >95% of PGC-1β overexpressing cells were viable (Fig. 7i). Interestingly, under these conditions, H_2_O_2_-induced endogenous transcript levels of *PCC-1α* and *PGC-1β* by 8.5 and 1.8-fold, respectively, and *Sod2* transcript levels by 1.6-fold (Supplementary Fig. 7). Moreover, PGC-1β overexpression induced *Sod2* transcript levels by 2.2-fold, but did not affect *PGC-1α* expression (Supplementary Fig. 7). In agreement with these results, electroporation of tibialis anterior muscle of PGC-1β^(i)skm−/−^ mice with a PGC-1β expression vector restored Sod2 transcript and protein levels (Fig. 7j,k and Supplementary Fig. 10c) and decreased ROS levels (Fig. 7l). Thus, PGC-1β enhances Sod2 expression in skeletal muscles and protects them from oxidative stress.

Note that even though oxidative stress was increased in PGC-1β^(i)skm−/−^ skeletal muscles, their mass was similar to that of control mice up to 1.5 years of age (Supplementary Fig. 8a). Moreover, histological analyses revealed no sign of muscle cellular infiltration and central nuclei, indicative of muscle damage or regeneration (Supplementary Fig. 8b), and the transcript levels of muscle regeneration markers (for example, myogenin, Pax7, Pax3, Serca 2 and sarcolipin) were similar in gastrocnemius of 1.5-year-old PGC-1β^(i)skm−/−^ mice and control mice, and those of embryonic MHC were undetectable in both cases (Supplementary Fig. 8c and data not shown).

**Figure 8:**
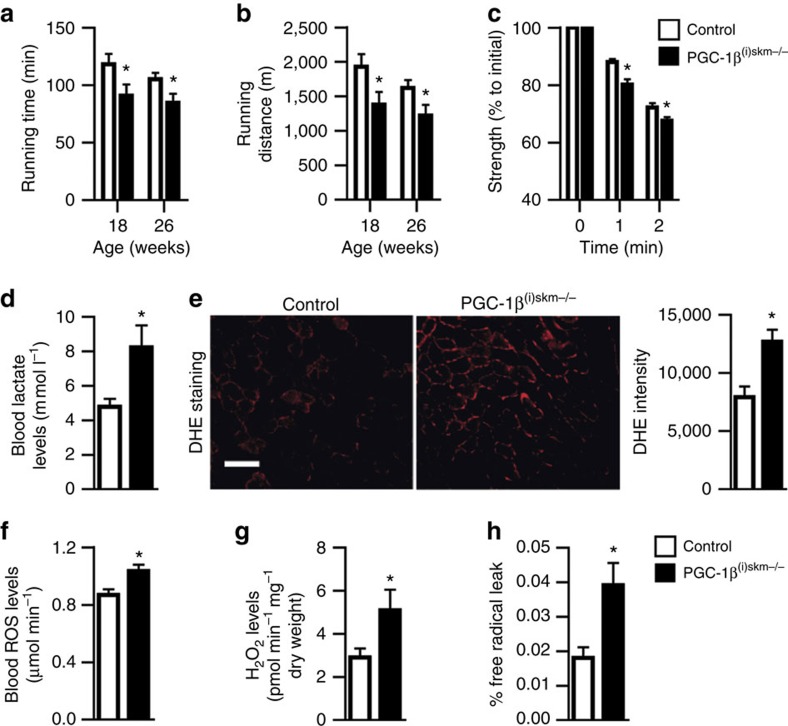
Oxidative stress in muscles of PGC-1β^(i)skm−/−^ mice after exercise. (**a**,**b**) Treadmill running time (**a**) and distance (**b**) of 18 and 26-week-old control and PGC-1β^(i)skm−/−^ mice subjected to run until exhaustion (*n*=12). (**c**) Fatigue resistance of tibialis anterior muscle of 18-week-old control and PGC-1β^(i)skm−/−^ mice to contractions (*n*=6). (**d**) Blood lactate levels in 26-week-old exercised control and PGC-1β^(i)skm−/−^ mice determined by a lactate pro-LT 1710 device (*n*=9). (**e**) DHE staining (left panel) and quantification of staining intensity (right panel) in gastrocnemius muscle of 26-week-old exercised control and PGC-1β^(i)skm−/−^ mice (*n*=9). Scale bar, 100 μm. (**f**) EPR quantification of ROS levels in blood of 26-week-old exercised control and PGC-1β^(i)skm−/−^ mice (*n*=9). (**g**) H_2_O_2_ levels determined by Amplex Red, in gastrocnemius muscle of 26-week-old exercised control and PGC-1β^(i)skm−/−^ mice (*n*=9). (**h**) Free-radical leak in gastrocnemius muscle of 26-week-old exercised control and PGC-1β^(i)skm−/−^ mice (*n*=9). Data are represented as mean±s.e.m. *P<0.05, Student's *t*-test.

To further determine the impact of myofibre PGC-1β deficiency on skeletal muscle function, 18 and 26-week-old mice were subjected to an exercise test. PGC-1β^(i)skm−/−^ mice were less effective at running than control mice (Fig. 8a,b). Moreover, *in situ* fatigue resistance was 10% lower in tibialis anterior muscle of 18-week-old mutant mice than in age-matched control mice (Fig. 8c), thus demonstrating that PGC-1β controls muscle contractility cell autonomously in skeletal muscles.

As skeletal muscle contractions enhance free-radical production^23^,^24^,^25^, we also evaluated the impact of PGC-1β ablation on exercise-induced oxidative stress. To this end, 26-week-old control and PGC-1β^(i)skm−/−^ mice were subjected to run on a treadmill until PGC-1β^(i)skm−/−^ mice were exhausted. Blood lactate levels were 1.8-fold higher in mutants than in controls (Fig. 8d), in agreement with earlier anaerobic glycolysis for ATP production due to mitochondrial dysfunction. Moreover, intracellular ROS levels were 1.6-fold higher in gastrocnemius and soleus muscles of mutant mice after exercise compared with control mice (Fig. 8e and Supplementary Fig. 9a), and total ROS levels in blood were increased by 1.3-fold in mutants (Fig. 8f). H_2_O_2_ levels were higher in gastrocnemius and soleus of mutant mice than of control mice (Fig. 8g and Supplementary Fig. 9b), and free-radical leak was twofold higher in gastrocnemius of PGC-1β^(i)skm−/−^ mice than of control mice at post-exercise condition (Fig. 8h). Note that even though ROS levels in gastrocnemius muscle of control mice were increased by 1.5-fold after running exercise, *PGC-1β* transcript levels remained unchanged (Supplementary Fig. 9c,d), thus indicating that physiological levels of ROS do not induce PGC-1β expression.

Taken together, these results show that myofibre PGC-1β is instrumental not only to coordinate the expression of genes controlling mitochondrial structure and oxidative phosphorylation to limit free-radical leak through the mitochondrial respiratory chain, but also to enhance the expression of the mitochondrial anti-oxidant enzyme Sod2, to lower ROS levels.

## Discussion

Analyses of mice in which PGC-1α and/or PGC-1β are ablated in the germ line or at early time in skeletal muscles led to controversies about their role in skeletal muscles^33^,^34^,^41^. Thus, to investigate the role of PGC-1β in skeletal muscle fibre maintenance, and bypass possible compensatory mechanisms that might occur during fibre formation, we generated PGC-1β^(i)skm−/−^ mice, in which PGC-1β is selectively ablated in skeletal myofibres at adulthood. As PGC-1α expression was unchanged in mutant muscles, even several months after PGC-1β ablation, the selective role of PGC-1β could be evaluated. We show that lack of PGC-1β in mature myofibres affects neither myofibre MHC composition in adult mice, nor muscle strength. Thus, these results, taken together with previous muscle loss-of-function studies of PGC-1β alone or in combination with PGC-1α (ref. 33), demonstrate that PGC-1β is dispensable for muscle fibre type determination and maintenance. However, even though the number of mitochondria was similar in myofibres of control and PGC-1β^(i)skm−/−^ mice, their size was decreased in mutants. About 7% of the mitochondria displayed cristae disruption and/or decreased matrix density 1 month after PGC-1β ablation, and up to 30% 2 months later. Moreover, mitochondrial respiration was decreased and endurance exercise performance impaired, thus demonstrating that PGC-1β controls mitochondrial activity in mature myofibres. A similar disconnection of mitochondrial content and activity was previously observed in skeletal muscles of mice in which PGC-1α and PGC-1β null alleles are induced during fibre formation^33^. The reason why mitochondrial size and cristae density were unaffected in such compound mice^33^ is however unclear, as skeletal muscles of hypomorphic PGC-1α mice in which PGC-1β is ablated during fibre formation also contain smaller mitochondria^34^. Moreover, the reduced number of myofibre mitochondria in the compound mice analysed by Zechner *et al.*^34^ but not in those investigated by Rowe *et al.*^33^ is surprising. Whether these discrepancies result from different efficiencies or timing of PGC-1α or PGC-1β ablation or variations of their genetic background remains to be determined. In any event, our results demonstrate that physiological levels of PGC-1α cannot fully compensate for the lack of PGC-1β, and that PGC-1β selectively controls mitochondrial size and function in mature myofibres.

Muscle mitochondrial dysfunction and reduced PGC-1α and PGC-1β levels in skeletal muscle have been proposed to play a causal role in the pathogenesis of insulin resistance and type 2 diabetes^38^,^39^,^42^,^43^,^44^,^45^,^46^. However, our results show that PGC-1β deficiency in skeletal myofibres does not alter glucose homeostasis of mice fed a regular chow or a HFD, even though their muscle mitochondria exhibit structural and functional abnormalities. These results are in agreement with a previous study showing that mitochondrial derangement, induced by muscle PGC-1 deficiency, does not lead to insulin resistance^34^. Our results also further extend the number of reports showing that muscle mitochondrial dysfunction is insufficient to induce insulin resistance (for reviews, see refs 47, 48, 49).

Our results reveal that the expression of numerous nuclear genes encoding proteins required for mitochondrial fatty-acid oxidation, citric cycle, oxidative phosphorylation, ROS scavenging and for mitochondrial import, translational machinery and fusion was decreased by 10–30% at early time after ablation of PGC-1β in myofibres. In addition, at later time the transcript levels of the nuclear-encoded mitochondrial transcription factors *Tfb1* and *Tfb2* were decreased, thereby impairing the expression of mitochondria encoded genes, further increasing mitochondrial defects. The transcriptional alterations induced by PGC-1β deficiency led to increased free-radical leak of the mitochondrial respiratory chain and decreased Sod2 levels, thereby enhancing oxidative stress. Note that even though high levels of oxidative stress (for example, in H_2_O_2_-treated myotubes) induced the transcript levels of PGC-1α and to a lesser extend those of PGC-1β, in agreement with previous experiments performed in non-muscle cells^9^, those induced by exercise running did not affect PGC-1β levels. As PGC-1α has been shown to stimulate the anti-oxidant defence system in various cultured cell types, and to protect neuronal, myocardial and vascular endothelial cells from oxidative damage^9^,^10^,^11^,^50^, PGC-1α might also contribute to control ROS levels in mature myofibres. In any event, our results show that myofibre PGC-1β is a major regulator of mitochondrial oxidative capacity to generate high levels of ATP production, and of anti-oxidant defence.

Strikingly, among the top 80 downregulated genes in PGC-1β-deficient myofibres, >10 (for example, *Cox5b*, *Cycs*, *Fh1*, *Mfn2*, *Mrpl47*, *Ndufa5*, *Ndufs1*, *Ndufs3*, *Ndufv1*, *Sdhb*, *Sdhd*, *Suclg1* and *Sod2*) contain binding sites for ERRs, orphan nuclear receptors involved in various aspects of energy homeostasis, and known to be coactivated by both PGC-1α and β (refs 2, 51, 52, 53, 54). In addition, several contain binding sites for GABPA, a protein which heterodimerizes with GABPB to form the NRF-2 (ref. 55), known to induce the expression of a number of nuclear genes specifying mitochondrial respiratory function^56^, and to be coactivated by PGC-1α (for example, *Cox4i1* and *Cox5b*)^54^. Thus, it is likely that PGC-1β coordinates the expression of many nuclear genes involved in myofibres, via ERRs and NRF-2. However, even though the nuclear genes encoding the mitochondrial transcription factors TFB1 and TFB2 also contain NRF-2-binding sites^56^ and were induced by PGC-1β overexpression, their transcript levels were reduced in PGC-1β-deficient skeletal muscles at later time than those of other nuclear-encoded mitochondrial proteins, indicating a higher messenger stability, or a lower dependency on PGC-1β coregulator activity. A motif search analysis of the −3 kb to+2 kb promoter region of the 80 most downregulated genes in PGC-1β^(i)skm−/−^ skeletal muscles, using the MEME bioinformatics tool^57^, revealed that all of them contain putative response elements for ERR, and 50 of them contained putative NRF-2-binding sites. Surprisingly, even though NRF-1 has been shown to be an important transcriptional regulator of genes involved in mitochondrial function^58^, only Cox5b contains a known NRF-1-binding site, and our motif search did not reveal any additional gene containing such a binding site. Thus, even though ERRs, NRF-1 and NRF-2 are known to be coregulated by PGCs (ref. 59), ERRs and NRF-2 appear to be key targets of PGC-1β in skeletal muscles.

Interestingly, PGC-1β expression decreases with age in human and mouse muscles^45^ (TGR, GL and DM, unpublished data), and exercise associated with intake of the polyphenol resveratrol increases PGC-1β expression in skeletal muscle of senescence-accelerated mice^60^. Moreover, we have recently shown that red wine polyphenols enhance PGC-1β expression in aging skeletal muscle, and prevent aging-related impairment in skeletal muscle mitochondrial function^61^. In addition, this study shows that mitochondrial activity and ROS scavenging in skeletal muscles of PGC-1β^(i)skm−/−^ mice can be enhanced by PGC-1β. Thus, induction of PGC-1β expression and/or activity in muscles might open new avenues to prevent or delay muscle impairments caused by mitochondrial dysfunction or ageing.

## Methods

### Generation of PGC-1β^(i)skm−/−^ mice

A 15-kb-targeting vector, encompassing PGC-1β exons 4–6 in which exon 5 encoding one of the three NR boxes of the nuclear receptor interacting domain was flanked by LoxP sites, was PCR generated (Fig. 1) and electroporated into embryonic stem (ES) cells. Targeted ES cells were injected into C57BL/6 blastocysts which were implanted in pseudopregnant females. Chimeric males were bred with flippase (FLP)-expressing females, to generate heterozygous PGC-1β^L2/+^ mice (bearing one PGC-1β L2 allele and one wild-type (WT; +) allele), which were backcrossed for 10 generations on C57BL/6 mice. PGC-1β^L2/+^ mice were bred with C57BL/6 HSA-Cre-ER^T2(tg/0)^ transgenic mice that express the tamoxifen-dependent Cre-ER^T2^ recombinase under the control of the HSA regulatory elements^35^, and their offsprings were intercrossed to generate HSA-Cre-ER^T2(tg/0)^/PGC-1β^L2/L2^ pre-mutant mice and HSA-Cre-ER^T2(0/0)^/PGC-1β^L2/L2^ control mice. Pre-mutant male mice and sex-matched control littermates were intraperitoneally injected with tamoxifen (1 mg per day, for 5 days) at 7 weeks of age.

Mice were genotyped by PCR amplification of genomic DNA extracted from tail biopsies using the DirectPCR extraction kit (Viagen, cat # 102-T) with PGC-1β primers 1 and 2, and Cre-ER^T2^ primers (Supplementary Table 1). PCR amplification with GAPDH primers (Supplementary Table 1) was used to generate an internal control DNA segment. Cre-mediated excision of the floxed PGC-1β DNA segments was determined by PCR amplification of genomic DNA isolated from mouse tissues using PGC-1β primers 1 and 3 (Supplementary Table 1).

### Mice care

Mice were maintained in a temperature and humidity controlled animal facility, with a 12-h light/dark cycle. Standard rodent chow (2,800 kcal kg^−1^, Usine d'Alimentation Rationelle, Villemoisson-sur-Orge, France) and water were provided ad libitum. HFD containing 4,056 kcal kg^−1^ (fat: 1,600 kcal kg^−1^ and sucrose: 1,600 kcal kg^−1^; Research Diets, New Brunswick, New Jersey) was given to mice at 5 weeks of age. Breeding and maintenance of mice were performed in the accredited IGBMC/ICS animal house (A67-218-37 notification of 16/10/2013), in compliance with French and EU regulations on the use of laboratory animals for research, under the supervision of D.M. who holds animal experimentation authorizations from the French Ministry of agriculture and Fisheries (N°67-209 and A 67-227). All animal experiments were approved by the Ethical committee Com'Eth (Comité d'Ethique pour l'Expérimentation Animale, Strasbourg, France). Animals were killed by cervical dislocation and tissues were immediately collected, weighed, and frozen in liquid nitrogen or processed for biochemical and histological analysis.

### Skeletal muscle electroporation

Gastrocnemius and tibialis anterior muscles of isofluorane anaesthetized mice were intra-muscularly injected with 20 μg of hyaluronidase (Sigma Aldrich) and 5 μg of a PGC-1β expression vector (#1026, addgene) 1 h later. Muscles were electroporated by applying 20 pulses of 100 V cm^−1^ for 20 ms using a CUY21 Edit System (Nepagene, Japan). The controlateral leg was electroporated with 5 μg of pcDNA3 vector, as control.

### Food consumption

Food pellets (150 g) were delivered to individually housed mice, and weighed after 1 week. Weekly food consumption was calculated by subtracting the final from the initial pellet weight.

### Blood analysis

Blood was collected from retro orbital sinus after a 6-h fast that started at the beginning of the light cycle. Serum glucose and insulin levels were determined with an Olympus AU400 analyzer (Olympus SA, Rungis, France) and a mouse ultra sensitive insulin ELISA kit (Mercodia AB, Uppsala, Sweden), respectively. Blood lactate was measured using a lactate pro-LT 1710 device (ARKRAY)^62^.

### Glucose tolerance and insulin sensitivity tests

Intraperitoneal glucose tolerance tests were performed on mice fasted for 14 h (overnight fast). After measurement of the basal glucose level on blood collected from the tail vein (time 0), mice were intraperitoneally injected with a 20% glucose solution in sterile saline (0.9% NaCl) at a dose of 2 g glucose per kg body weight. Blood was collected from the tail vein after 15, 30, 45, 60, 90, 120, 150 and 180 min for glucose determination. Blood glucose levels were determined with an Accu-Chek Active blood glucometer (Roche, France). For insulin sensitivity tests (IPIST), 6 h fasted mice were intraperitoneally injected with porcine insulin (0.5 U kg^−1^; Sigma), and blood was collected at 15, 30, 60, and 90 min, and glucose levels were measured.

### Body lean and fat content

Body lean and fat content were recorded in anaesthetized mice by Dual Energy X-ray Absorptiometry (PIXIMUS, GE Medical Systems, Buc, France) according to the manufacturer's instructions.

### Grip strength

A Grip Strength Meter (Bioseb) was used to measure forelimb and hindlimb grip strength. The test was repeated three consecutive times within the same session, and the mean value was recorded as the maximal grip strength for each mouse.

### Exercise performance test

Exercise performance tests were performed on a motorized treadmill (LE 8700; Control Panlab Instrument). Mice were accustomed to treadmill running for 3 days (10 min runs in the morning and in the afternoon; day 1, no incline and 15 cm s^−1^ belt speed in the morning and 20 cm s^−1^ in the afternoon; day 2, 10° incline and 20 cm s^−1^ belt speed in the morning and 25 cm s^−1^ in the afternoon; day 3, 15° incline and 25 cm s^−1^ belt speed). At day 4, following a warm up phase of 10 min (0° incline, 25 cm s^−1^ belt speed), animals were run for 50 min at a 15° incline and a belt speed of 25 cm s^−1^, followed by 30 min at 20° incline and 28 cm s^−1^. Thereafter, the belt speed was increased for 3 cm s^−1^ every 30 min, until mice were exhausted.

### Contractile measurements

Mice were anaesthetized using pentobarbital (60 mg kg^−1^ i.p.), and body temperature was maintained at 37 °C using radiant heat. The knee and foot were fixed with pins and clamps and the distal tendon of tibialis anterior muscle was attached to a lever arm of a servomotor system (305B, Dual-Mode Lever, Aurora Scientific) using a silk ligature. The sciatic nerve was proximally crushed and distally stimulated by a bipolar silver electrode using supramaximal square wave pulses of 0.1 ms duration. Absolute maximal force (P0) generated during isometric contractions in response to electrical stimulation (frequency of 75–150 Hz, train of stimulation of 500 ms) was determined at L0 (length at which maximal tension was obtained during the tetanus). The fatigue resistance protocol consisted of 40 contractions (100 Hz for 300 ms, evoked once every second), and the force produced at the end of the protocol was measured (% of initial force). Muscle weight was determined to calculate specific maximal force.

### Histological analysis

Muscle tissue was immediately frozen after dissection in liquid nitrogen-cooled isopentane.

For NADH-tetrazolium reductase staining, 10 μm cryosections were incubated in 0.2 M Tris-HCl pH 7.4, containing 1.5 mM NADH and 1.5 mM nitrobluetetrazolium (NBT) for 15 min at 55 °C and washed with three exchanges of deionized H_2_O. The unbound NBT were removed from the sections with three exchanges each of 30, 60 and 90% acetone solutions in increasing and then decreasing concentration. The sections were finally rinsed several times with deionized water and mounted with aqueous mounting medium^63^.

For SDH staining, 10 μm cryosections were incubated for 1 h in 20 mM potassium dihydrogen phosphate, 76 mM di-sodium hydrogen phosphate, 5.4% sodium succinate and 0.02% NBT, washed in Dulbecco's PBS for 5 min 3 times. The sections were later postfixed in 10% buffered formalin solution for 10 min, rinsed in 15% ethanol for 5 min two times and mounted with aqueous mounting medium^64^.

For immunohistochemistry, 10 μm cryosections were rehydrated and fixed with 4% paraformaldehyde for 10 min. Fixed sections were blocked with 5% normal goat serum (NGS, VS-1000, Coger Reactifs Et Produits De La Recherche) in PBS containing 0.1% Triton-X100 for 1 h at room temperature, and were incubated overnight at 4 °C with anti-MHC1 (M 8421, Sigma, 1/2000), -MHC2A, -MHC2X and -MHC2B antibodies (cell culture supernatant from SC-71, 6H1 and BF-F3 hybridoma, respectively, obtained from the German collection of microorganisms and cell cultures). Slides were then incubated with secondary antibodies (GAM-Alexa 488 (IgG), A-21121, Invitrogen, 1/400 for MHC1 and 2A, and GAM-Alexa 488 (IgM), A-21042, Invitrogen, 1/400 for MHC2X and 2B) for 1 h at room temperature. Sections were mounted in aqueous medium with 4′,6-diamidino-2-phenylindole (DAPI) (SIGMA, D-9542, 1/1000). Between each step, sections were washed with PBS, 0.1% Triton-X100. Stained fibres were quantified manually using ImageJ.

Ultrastructural analyses were performed as described^65^. Skeletal muscle samples were fixed by immersion in 2.5% glutaraldehyde and 2.5% paraformaldehyde in cacodylate buffer (0.1 M, pH 7.4) and washed in cacodylate buffer for 30 min and kept at 4 °C. Post-fixation was performed with 1% osmium tetraoxide in 0.1 M cacodylate buffer for 1 h at 4 °C and dehydration through graded alcohol (50, 70, 90 and 100%) and propylene oxide for 30 min each. Samples were oriented longitudinally and embedded in Epon 812. Ultrathin sections were cut at 70 nm and contrasted with uranyl acetate and lead citrate, and examined at 70 kv with a Morgagni 268D electron microscope. Images were captured digitally by a Mega View III camera (Soft Imaging System). Mitochondria density and cross-section area were determined from the electron micrographs using ImageJ software. Normal and abnormal mitochondria were counted in a blinded fashion on 10 gastrocnemius muscle sections per mouse, from five control mice and six mutant mice (∼500 mitochondria).

### Fibre CSA measurements

Muscle cross-sections were immunohistochemically stained for dystrophin (ab15277, Abcam, 1/100) to mark the sarcolemma surrounding each fibre, and CSA was quantified using the FIJI image-processing software.

### Mitochondria respiration analysis

Muscles were collected and placed in S solution containing 2.77 mM CaK_2_ ethylene glycol tetraacetic acid (EGTA), 7.23 mM K_2_EGTA, 6.56 mM MgCl_2_, 20 mM taurine, 0.5 mM DTT, 50 mM potassium-methane-sulfonate (160 mM ionic strength) and 20 mM imidazole (pH 7.1), 5.7 mM Na_2_ATP and 15 mM creatine-phosphate. Fibres were separated under a binocular microscope in solution S at 4 °C and permeabilized for 30 min in solution S with 50 μg ml^−1^ of saponin. The permeabilized fibres were later placed for 10 min in solution R containing 2.77 mM CaK_2_EGTA, 7.23 mM K_2_EGTA, 6.56 mM MgCl_2_, 20 mM taurine, 0.5 mM DTT, 50 mM potassium-methane-sulfonate (160 mM ionic strength), 20 mM imidazole (pH 7.1), 5 mM glutamate, 2 mM malate, 3 mM phosphate and 2 mg ml^−1^ fatty acid-free bovine serum to wash out adenine nucleotides and creatine-phosphate, and then transferred to a 3-ml water-jacketed oxygraphic cell (Strathkelvin Instruments, Glasgow, UK) equipped with a Clark electrode. V_0_ was measured in the absence of fibres and substrate at 22 °C under continuous stirring. V_0_ corresponds to the rate of O_2_ consumption after addition of muscle fibres and glutamate–malate as substrate, to which V_0_ is subtracted. V_MAX_ (maximal respiration) corresponds to the rate of O_2_ consumption after addition of 2 mM of ADP as phosphate acceptor, to which V_0_ is subtracted. After measurement, fibres were dried, and respiration rates were expressed as micromoles of O_2_ per minute per gram dry weight.

### Quantification of mitochondrial and nuclear DNA

Gastrocnemius muscle was digested overnight with Proteinase K, and DNA was extracted by phenol–chloroform method. Mitochondrial DNA content was determined by quantitative PCR analysis. The levels of cytochrome oxidase 2 (Cox2; mitochondrial DNA) were normalized to the levels of fatty-acid synthase (Fas; genomic DNA) to evaluate the mitochondria number. The primer sequences are given in Supplementary Table 1.

### DHE staining for ROS measurement

Muscle cryosections (10 μm) were incubated for 30 min at 37 °C with 2.5 μM DHE in PBS. DHE produces a red fluorescence when oxidized to ethidium bromide by superoxide anions^66^. After staining, the sections were rinsed, air-dried, mounted in Vectashield (Vector Laboratories, Burlingame, CA). Slides were examined under a fluorescence microscope. The emission signal was recorded using a Zeiss 573–637 nm filter and the number of pixels per field was quantified.

### H_2_O_2_ production in permeabilized fibres

Permeabilized bundles were placed in ice-cold buffer Z containing 110 mM methane-sulfonate, 35 mM KCl, 1 mM EGTA, 5 mM K_2_HPO_4_, 3 mM MgCl_2_, 6 mM H_2_O, 0.05 mM glutamate and 0.02 mM malate with 0.5 mg ml^−1^ BSA (pH 7.1, 295 mosmol kg^−1^ H_2_O). H_2_O_2_ production was measured with Amplex Red (Invitrogen), which reacts with H_2_O_2_ in a 1:1 stoichiometry catalysed by HRP to yield the fluorescent compound, resorufin and a molar equivalent of O_2_. Resorufin has excitation and emission wavelengths of 563 and 587 nm, respectively, and is extremely stable once formed. Fluorescence was measured continuously with a Fluoromax 3 (Jobin Yvon) spectrofluorometer with temperature control and magnetic stirring. After a baseline (reactants only) was established, the reaction was initiated by adding a permeabilized fibre bundle to 600 μl of buffer Z. Buffer Z contained 5 mM Amplex Red, 0.5 U ml^−1^ HRP, 5 mM glutamate and 2 mM malate as substrates at 37 °C. At the end of each experiment, fibres were harvested and dried for 15 min at 150 °C. The results were reported in pmol min^−1^ mg^−1^ dry weight.

### Mitochondrial free-radical leak

H_2_O_2_ production and O_2_ consumption were measured in parallel in the same sample. This allowed the calculation of the fraction of electrons out of sequence which reduce O_2_ to ROS in the respiratory chain (the per cent of free-radical leak) instead of reaching cytochrome oxidase to reduce O_2_ to water^67^,^68^. Because two electrons are needed to reduce one molecule of O_2_ to H_2_O_2_, whereas four electrons are transferred in the reduction of one molecule of O_2_ to water, the per cent free-radical leak was calculated as the rate of H_2_O_2_ production divided by two times the rate of O_2_ consumption, and the result was multiplied by 100.

### Electron paramagnetic resonance

The experimental protocol for ROS detection in blood was adapted from ref. 69. Blood samples (∼20 μl) were collected from the tail vein, stored on ice for 30 min and mixed with 20 μl of spin probe CMH (1-hydroxy-3-methoxycarbonyl-2, 2, 5, 5-tetramethylpyrrolidine HCl)/heparin (400 μM per 100 U per ml) solution. The stock solution of CMH is 5 mM in 2 ml of Krebs-4-(2-hydroxyethyl)-1-piperazineethanesulfonic acid (HEPES) buffer containing 25 μM deferoxamine methane-sulfonate salt (DF) chelating agent and 5 mM sodium diethyldithio-carbamatetrihydrate (DETC) at pH 7.4. Immediately after 40 μl of the obtained solution was introduced in the glass electron paramagnetic resonance (EPR) capillary tube (Noxygen Science Transfer & Diagnostics, Germany), that was placed in the e-scan spectrometer (Bruker, Germany) for data acquisition. Sample temperature was firstly stabilized and then kept at 37 °C by the Temperature & Gas Controller ‘Bio III' unit, interfaced to the spectrometer. ROS detection was conducted using BenchTop EPR spectrometer E-SCAN under the following EPR settings: centre field *g*=2.011; field sweep 60G; microwave power 20 mW; modulation amplitude 2G; conversion time 10.24 ms; time constant 40.96 ms, number of scans: 10. The EPR signal is proportional to the unpaired electron numbers and could, in turn, be transformed in absolute produced micromoles (μmol min^−1^).

### Cell culture

C_2_C_12_ myoblasts (ATTC, CRL-1772) were cultured following supplier's recommendations. Myoblast were electroporated (Gene pulser II, Biorad) with the empty-vector pcDNA3 or a PGC-1β expression vector (#1026, addgene), following the supplier's protocol. After 3 days of differentiation, cells were treated 6 h with 1.5 mM H_2_O_2_ (Sigma Aldrich, H3410). Cellular viability was assessed by trypan blue.

### Protein preparation and analysis

Muscles were grounded at 4 °C in radioimmunoprecipitation assay (RIPA) buffer (50 mM Tris pH 7.5, 1% Nonident P40, 0.5% sodium deoxycholate, 0.1% SDS, 150 mM NaCl, 5 mM EDTA, 1 mM phenylmethanesulphonylfluoride (PMSF) and protease inhibitor cocktail (45 μg ml^−1^, 11 873 580 001, Roche)) with a potter. Sod activity was determined by a colorimetric assay (19160, SOD determination kit, Sigma Aldrich) according supplier's protocol. Homogenates (50 μg of protein) were electrophoresed on 12% polyacrylamide gels. Proteins were electroblotted to Hybond nitrocellulose membranes (Amersham Biosciences) and immunodetected using primary antibodies directed against PGC-1β (AF5656, R & D systems, 1/1,000), Ndufs3 (439200, invitrogen, 1/1,000), Sdhb (459230, invitrogen, 1/2,000), Cytochrome C (556433, BD Biosciences, 1/1,000), Sod2 (SOD-110E, Euromedex, 1/2,000) and Gapdh (MAB374, MILLIPORE UPSTATE CHEMICON, 1/10,000). Membranes were probed with secondary antibodies conjugated to HRP (Amersham Biosciences), which were revealed using an enhanced chemiluminescence detection system (Pierce, Rockford, IL, 1/10,000).

### Lipid peroxidation assay

Muscle homogenates of 100 μl were added to 200 μl of Trichloroacetic acid/thiobarbituric acid/HCl (15% TCA, 0.375% TBA, 0.25 N HCl) and boiled for 15 min in cryotubes. After centrifugation for 10 min at 2,500*g*, absorbance was read at 535 nm. The malondialdehyde (MDA) concentration of the sample was calculated using an extinction coefficient of 1.56 × 10^5^ M^−1^ cm^−1^.

### RNA extraction and analysis

Total RNA was isolated using TRIzol reagent (Invitrogen). cDNA was synthesized from 2 μg of RNA by reverse transcription using random primers and SuperScript II reverse transcriptase (Invitrogen, Life Technologies), according to the supplier's protocol. Quantitative RT-PCR analysis was performed with gene-specific primers using the QuantiTectTM SYBR Green PCR kit (Roche), according to supplier's protocol. Relative abundance of transcript levels was calculated after normalization to hypoxanthine-guanine phosphoribosyltransferase (*Hprt*). The primer sequences are given in Supplementary Table 1.

### Microarray analysis

Gene expression profiling was performed on total RNA isolated from gastrocnemius muscle of three 8-week-old control and mutant mice. Biotinylated single-strand cDNA targets were prepared, starting from 150 ng of total RNA, using the Ambion WT Expression Kit (Cat # 4411974) and the Affymetrix GeneChip WT Terminal Labeling Kit (Cat # 900671) according to Affymetrix recommendations. Following fragmentation and end-labelling, 1.9 μg of cDNA was hybridized for 16 h at 45 °C on GeneChip Mouse Gene 1.0 ST arrays (Affymetrix) interrogating 28,853 genes represented by ∼27 probes spread across the full length of the gene. The chips were washed and stained in the GeneChip Fluidics Station 450 (Affymetrix) and scanned with the GeneChip Scanner 3000 7G (Affymetrix) at a resolution of 0.7 μm. Raw data (CEL Intensity files) were extracted from the scanned images using the Affymetrix GeneChip Command Console (AGCC) version 3.2. CEL files were further processed with Affymetrix Expression Console software version 1.1 to calculate probe set signal intensities using Robust Multi-array Average (RMA) algorithms with default settings. Raw data were submitted to GEO datasets. Genes were considered to be downregulated if expression was ≤0.8-fold and upregulated if it was ≥1.2-fold compared with the respective control. Data sets were uploaded on the Database for Annotation, Visualization and Integrated Discovery (DAVID) for gene ontology analyses.

### Motif analysis

The motif discovery programme MEME (ref. 57) was used to identify motifs that are enriched with high frequency in an unbiased manner. The input consisted of 5,000 bp (3,000 bp upstream of transcriptional start site and 2,000 downstream of transcriptional start site) of the 80 most downregulated genes in PGC-1β^(i)skm−/−^ muscle. The motifs from MEME were queried against the JASPAR and UNIPROBE database using Tomtom^70^ to identify the best-matching known motifs.

### Data analysis

Data were collected blindly from mice, as investigators did not know their genotype. A minimum of six animals was used for inter-individual comparison, and four for intra-individual comparison. No inclusion/exclusion criteria, and no method of randomization were used for this study. Data are represented as mean±s.e.m. (s.e. of the mean). Statistical comparisons of data from two experimental groups were made by using a two-tailed Student's test and were considered to be statistically significant if *P*<0.05 and are indicated by an asterisk in the figures. The samples followed a normal distribution, and the variances were similar.

## Additional information

**Accesion code:** Gene expression data have been deposited in GEO datasets under accession code GSE73572.

**How to cite this article:** Gali Ramamoorthy, T. *et al.* The transcriptional coregulator PGC-1β controls mitochondrial function and anti-oxidant defence in skeletal muscles. *Nat. Commun.* 6:10210 doi: 10.1038/ncomms10210 (2015).

## Supplementary Material

Supplementary Information Supplementary Figures 1-10 and Supplementary Table 1
