# Supplementary material for: The transcriptional coregulator PGC-1β controls mitochondrial function and anti-oxidant defence in skeletal muscles
Source: Nat Commun. 2015 Dec 17;6:10210. doi: 10.1038/ncomms10210 (PMC4703903; doi:10.1038/ncomms10210)
Supplement: Supplementary Information — Supplementary Figures 1-10 and Supplementary Table 1 [file ncomms10210-s1.pdf]

Supplemental information

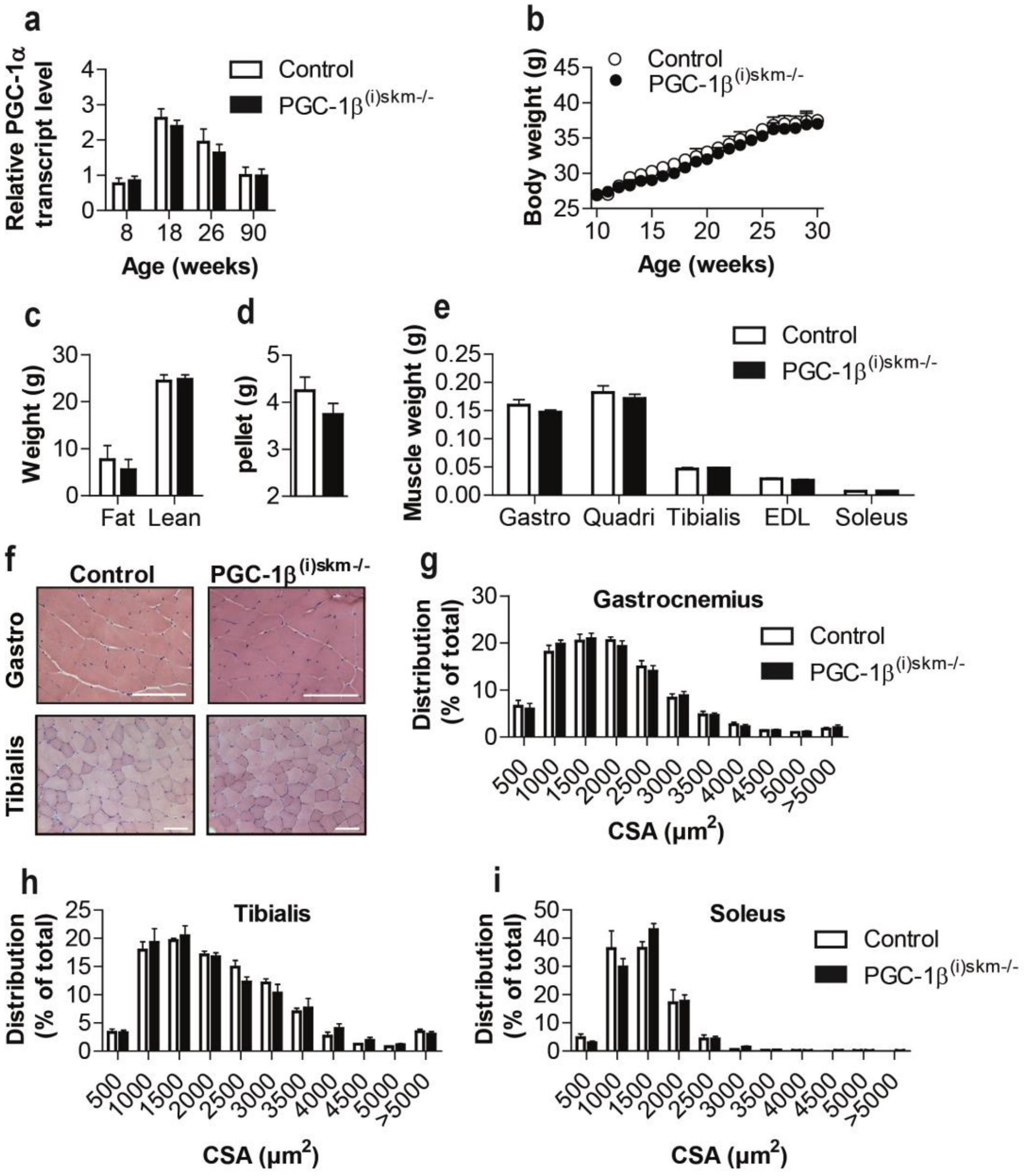

Supplementary Figure 1. Characterization of PGC-1β$^{(i)skm-/-}$ mice.

(a) PGC-1α transcript levels in gastrocnemius muscle of control and PGC-1β$^{(i)skm-/-}$ mice at the indicated age, determined by RT-qPCR (n=4). **(b)** Body weight of control and PGC-1β$^{(i)skm-/-}$ mice (n=11). **(c)** Lean and fat content of 26 week-old control and PGC-1β$^{(i)skm-/-}$ mice analyzed by DEXA scanning (n=7). **(d)** Food intake in 18 week-old control and PGC-1β$^{(i)skm-/-}$ mice (n=8). **(e)** Weight of gastrocnemius (Gastro), quadriceps (Quadri), tibialis anterior (Tibialis), Extensor digitorum longus (EDL) and soleus muscles from 26 week-old control and PGC-1β$^{(i)skm-/-}$mice (n=9). **(f)** Representative hematoxylin and eosin stained sections of gastrocnemius (Gastro) and tibialis muscles from 26 week-old control and PGC-1β$^{(i)skm-/-}$ mice (n=4). Scale bar: 100 µm. **(g-i)** Distribution of gastrocnemius **(g)**, tibialis anterior **(h)** and soleus **(i)** fiber cross section area (CSA) from 26 week-old control and PGC-1β$^{(i)skm-/-}$ mice (n=8). Data are represented as mean +/- s.e.m. *, $p<0.05$, student's t test.

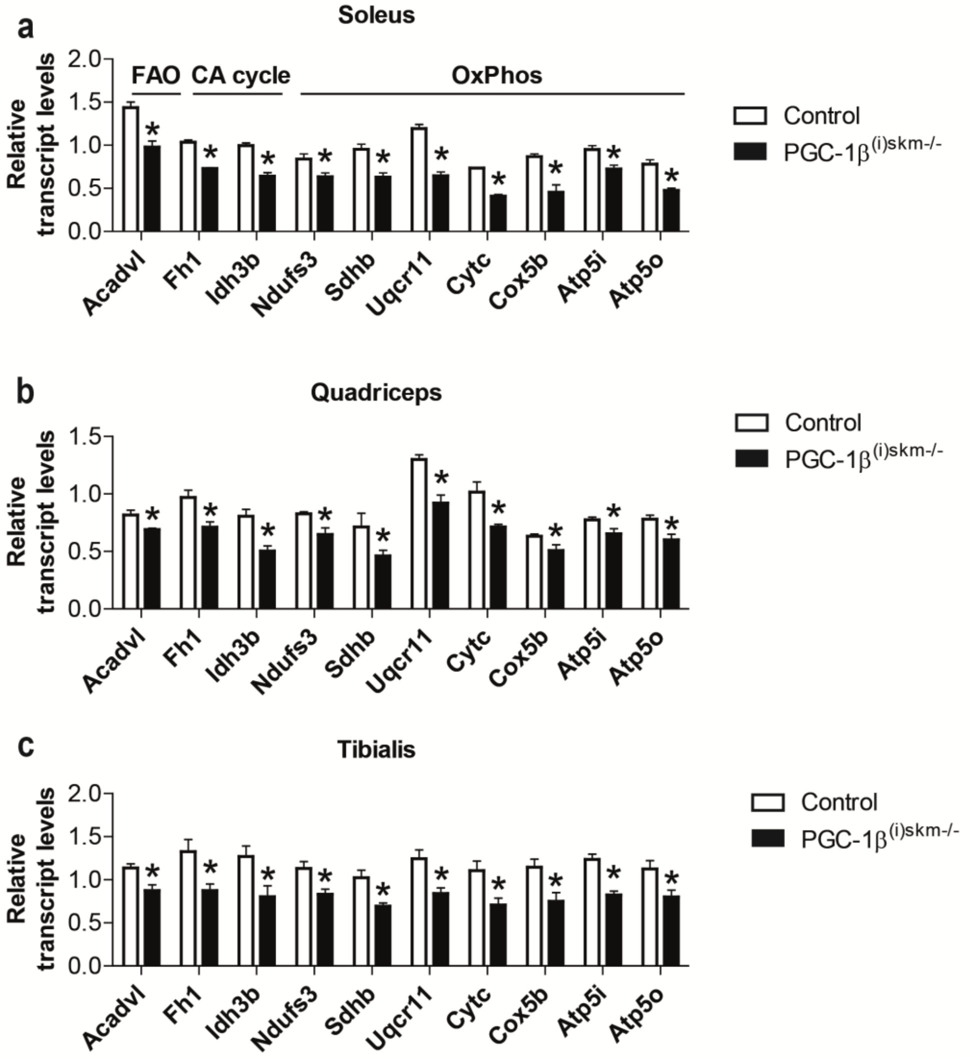

Supplementary Figure 2. Transcript levels of genes encoding proteins involved in energy metabolism in control and PGC-1β$^{(i)skm-/-}$ muscle. **(a-c)** Relative transcript levels of Acadvl (FAO, Fatty Acid Oxidation), Fh1 and Idh3b (CA, Citric Acid cycle), Ndufs3, Sdhb, Uqcr11, Cytc, Cox5b, Atp5i and Atp5o (OXPHOS, Oxidative phosphorylation) in soleus **(a)**, quadriceps **(b)** and tibialis anterior **(c)** of 8 week-old control and PGC-1β$^{(i)skm-/-}$ mice, determined by RT-qPCR (n=4). Data are represented as mean +/- s.e.m. *, $p<0.05$, student's t test.

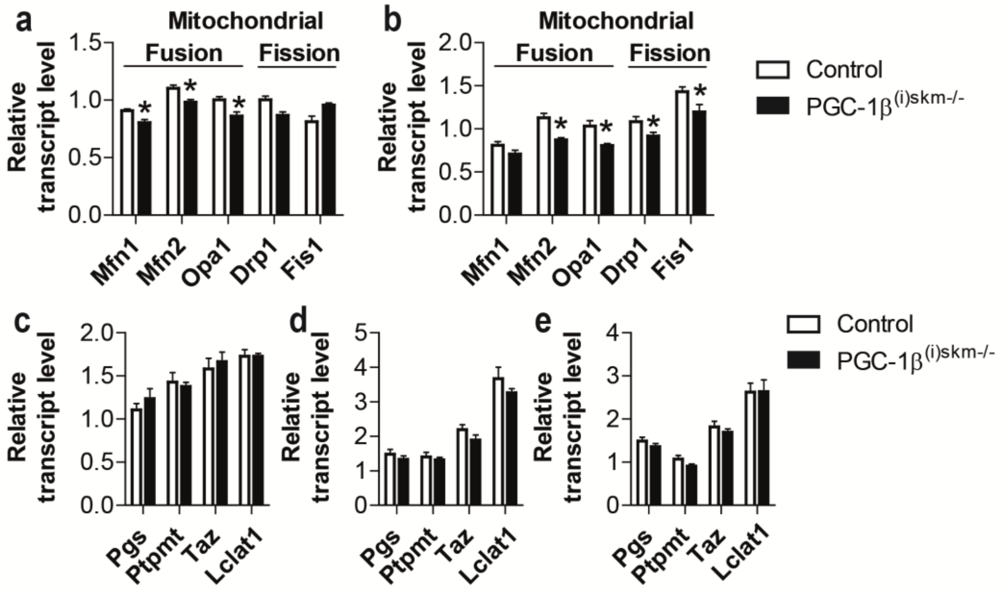

Supplementary Figure 3. Transcript levels of gene encoding proteins involed in mitochondrial dynamics and cardiolipin synthesis and remodelling. Relative transcript levels of Mfn1, Mfn2, Opa1, Drp1 and Fis1 in gastrocnemius muscle of 18 **(a)** and 26 **(b)** week-old control and PGC-1β $^{(i)skm-/-}$ mice, determined by RT-qPCR (n=8). **(c-e)** Relative transcript levels of Pgs, Ptpmt, Taz and Lclat1 in gastrocnemius muscle of 8 **(c)**, 18 **(d)** and 26 **(e)** week-old control and PGC-1β $^{(i)skm-/-}$ mice, determined by RT-qPCR (n=8). Data are represented as mean +/- s.e.m. *, $p<0.05$, student's t test.

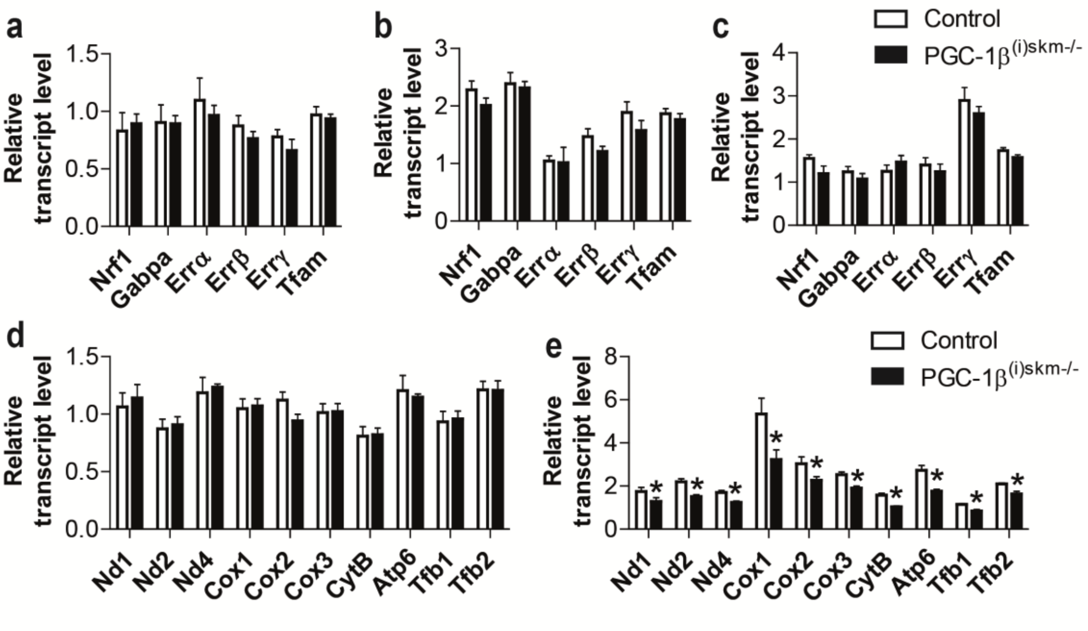

Supplementary Figure 4. Transcript levels of nuclear encoded transcription factors involved in mitochondrial biogenesis and of mitochondrial encoded proteins. Relative transcript levels of Nrf1, Gabpa, Errα, Errβ, Errγ and Tfam in gastrocnemius muscle of 8 **(a)**, 18 **(b)** and 26 **(c)** week-old control and PGC-1β$^{(i)skm-/-}$ mice, determined by RT-qPCR (n=8). **(d-e)** Relative transcript levels of Nd1, 2 and 4 (NADH deshydrogenase subunit 1, 2 and 4), Cox1, 2 and 3 (Cytochrome c oxidase subunit 1, 2 and 3), CytB (cytochrome B), Atp6 (ATP synthase 6), and of Tfb1 and Tfb2 in gastrocnemius muscle of 8 **(d)** and 26 **(e)** week-old control and PGC-1β$^{(i)skm-/-}$ mice, determined by RT-qPCR (n=8). Data are represented as mean +/- s.e.m. *, $p<0.05$, student's t test.

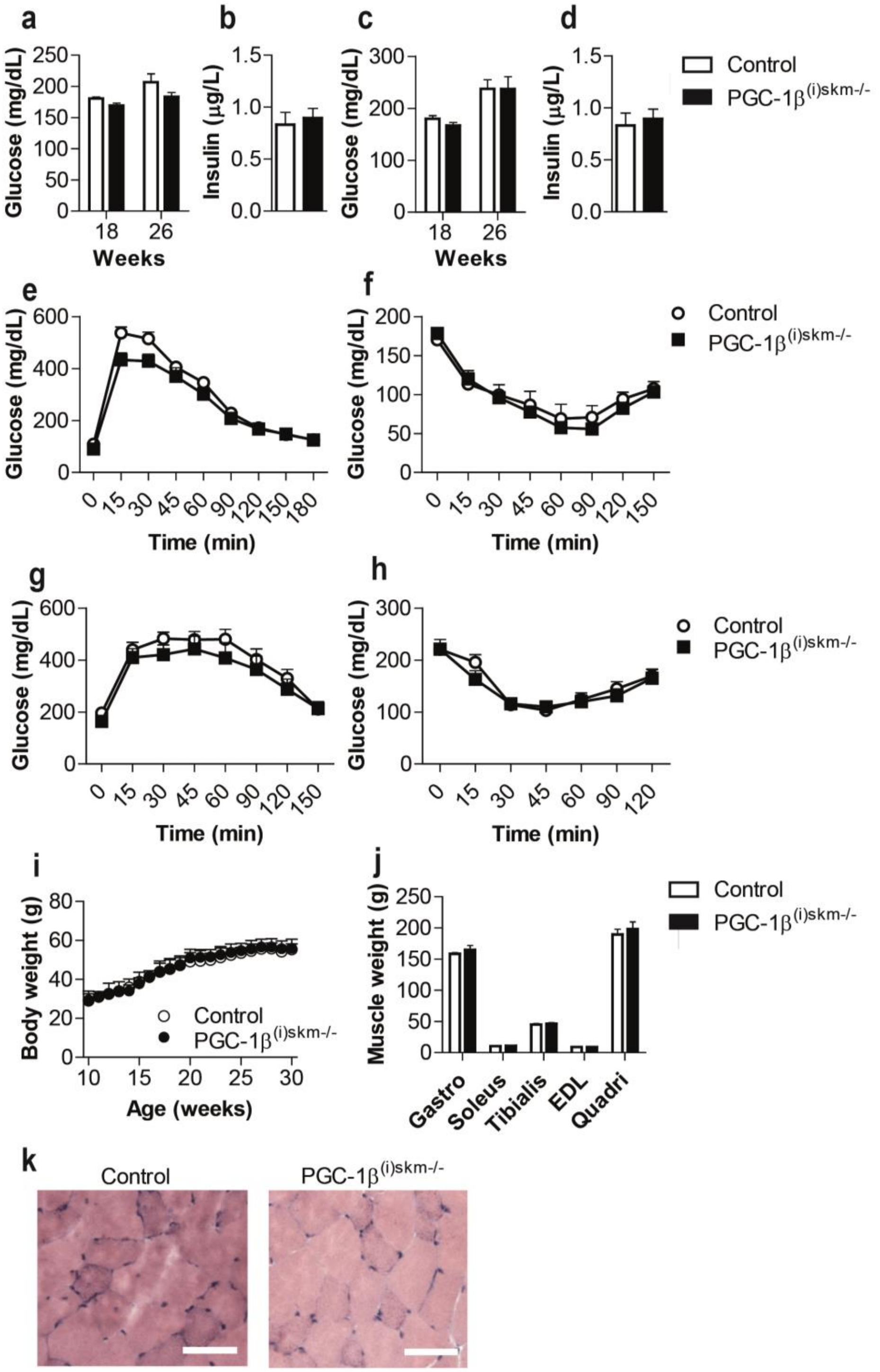

Supplementary Figure 5. Glucose homeostasis in PGC-1$\beta^{(i)skm-/-}$ mice fed a regular chow or a high fat diet. (a) Fasting serum glucose levels in 18 and 26 week-old control and PGC-1β $^{(i)skm-/-}$ mice (n=10) fed a regular chow diet, determined with an Olympus AU400 analyzer. **(b)** Fasting serum insulin levels in 26 week-old control and PGC-1β $^{(i)skm-/-}$ mice fed a regular chow diet, determined with a mouse ultra sensitive insulin ELISA kit (n=10). **(c)** Fasting serum glucose levels in 18 and 26 week-old control and PGC-1$\beta^{(i)skm-/-}$ mice fed high fat diet, determined with an Olympus AU400 analyzer (n=10). **(d)** Fasting serum insulin levels in 26 week-old control and PGC-1$\beta^{(i)skm-/-}$ mice fed high fat diet, determined with a mouse ultra sensitive insulin ELISA kit (n=10). Blood glucose levels, determined with an Accu-Chek Active blood glucometer during an intraperitoneal glucose tolerance test (IPGTT) **(e)** and an intraperitoneal insulin sensitive test (IPIST) **(f)**, in 26 week-old control and PGC-1$\beta^{(i)skm-/-}$ mice fed regular chow diet. (n=9). Blood glucose levels, determined with an Accu-Chek Active blood glucometer during an IPGTT **(g)** and an IPIST **(h)**, in 26 week-old control and PGC-1$\beta^{(i)skm-/-}$ mice fed high fat diet (n=9). **(i)** Body weight of control and PGC-1$\beta^{(i)skm-/-}$ mice fed high fat diet. Muscle mass **(j)** and representative hematoxylin and eosin stained sections of gastrocnemius muscle **(k)** from 26 weeks control and PGC-1$\beta^{(i)skm-/-}$ mice fed high fat diet (n=9). Scale bar: 100 µm. Data are represented as mean +/- s.e.m. *, $p<0.05$, student's t test.

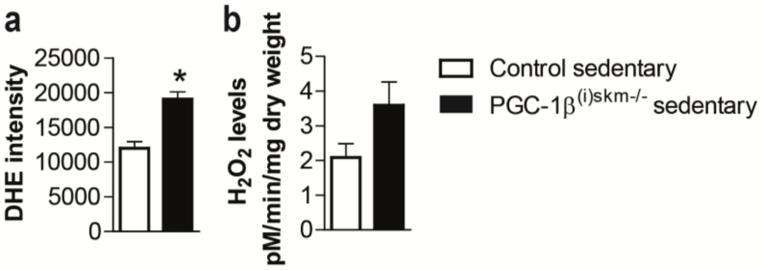

Supplementary Figure 6. Oxidative stress in soleus muscle of sedentary PGC-1β $^{(i)skm-/-}$ mice. **(a)** Dihydroethidium (DHE) staining intensity and **(b)** H_2O_2 levels determined by Amplex Red, in soleus muscle of 26 week-old sedentary control and PGC-1β$^{(i)skm-/-}$ mice (n=9). Data are represented as mean +/- s.e.m. *, $p<0.05$, student's t test.

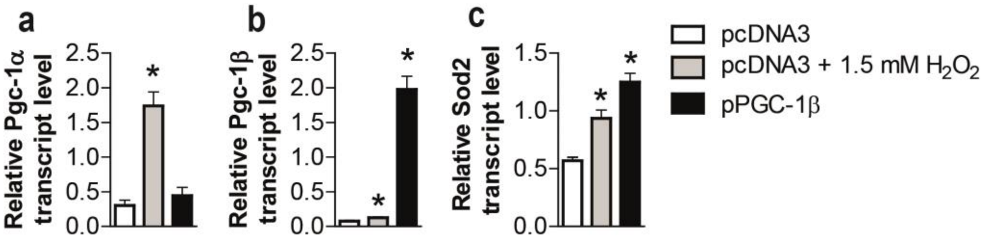

Supplementary Figure 7. Transcript levels in C_2C_{12} cells treated with H_2O_2 or transfected with a PGC-1β expression vector. Transcript levels determined by RT-qPCR of **(a)** PGC-1α, **(b)** PGC-1β and **(c)** Sod2 in C_2C_{12} cells electroporated with an empty vector (pcDNA3) and cultured 6 h in presence (grey bars) or in absence (white bars) of 1.5 mM of H_2O_2, and in untreated C_2C_{12} cells electroporated with a PGC-1β expression vector (pPGC-1β, black bars) (n=3). Data are represented as mean +/- s.e.m. *, $p<0.05$, student's t test.

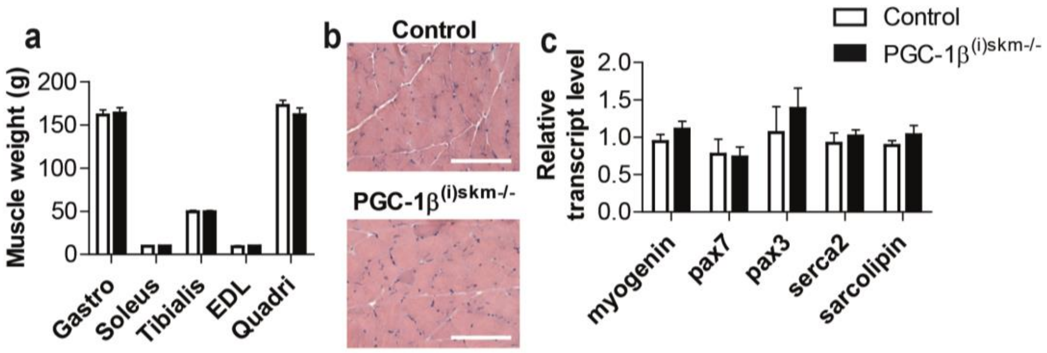

Supplementary Figure 8. Characterization of muscles in aged PGC-1β$^{(i)skm-/-}$ mice. (a) Muscle mass and **(b)** representative hematoxylin and eosin stained sections of gastrocnemius muscle from 90 week-old control and PGC-1β$^{(i)skm-/-}$ mice (n=3). Scale bar: 100 µm. **(c)** Transcript levels of genes encoding myofiber regeneration markers in tibialis muscle from 90 week-old control and PGC-1β$^{(i)skm-/-}$ mice, determined by RT-qPCR. (n=3). Data are represented as mean +/- s.e.m.

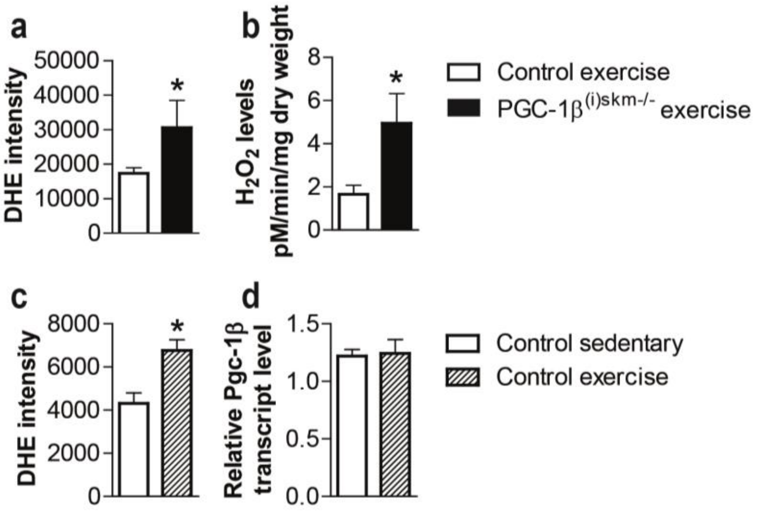

Supplementary Figure 9. Oxidative stress in muscles of PGC-1β$^{(i)skm-/-}$ mice after exercise. **(a)** Dihydroethidium (DHE) staining intensity in soleus muscle of exercised 26 week-old control and PGC-1β$^{(i)skm-/-}$ mice (n=9). **(b)** H_2O_2 levels determined by Amplex Red, in soleus muscle of exercised 26 week-old control and PGC-1β$^{(i)skm-/-}$ mice (n=9). **(c)** Dihydroethidium (DHE) staining intensity and **(d)** PGC-1β transcript levels, determined by RT-qPCR, in gastrocnemius muscle of 26 week-old sedentary and exercised control mice (n=9). Data are represented as mean +/- s.e.m. *, $p<0.05$, student's t test.

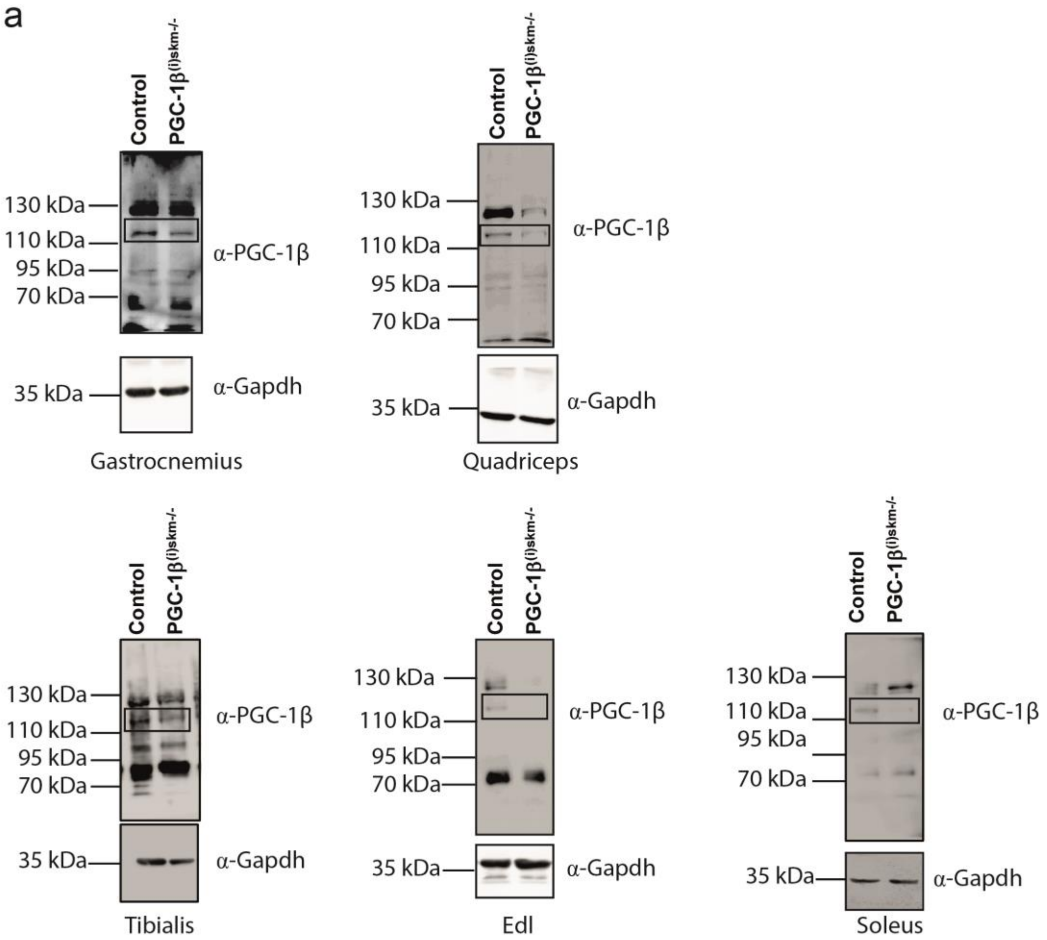

a
Control
PGC-1β(i)skm-/-
130 kDa
110 kDa
95 kDa
70 kDa
35 kDa
α-PGC-1β
α-Gapdh
Gastrocnemius
Quadriceps
Tibialis
Edl
Soleus

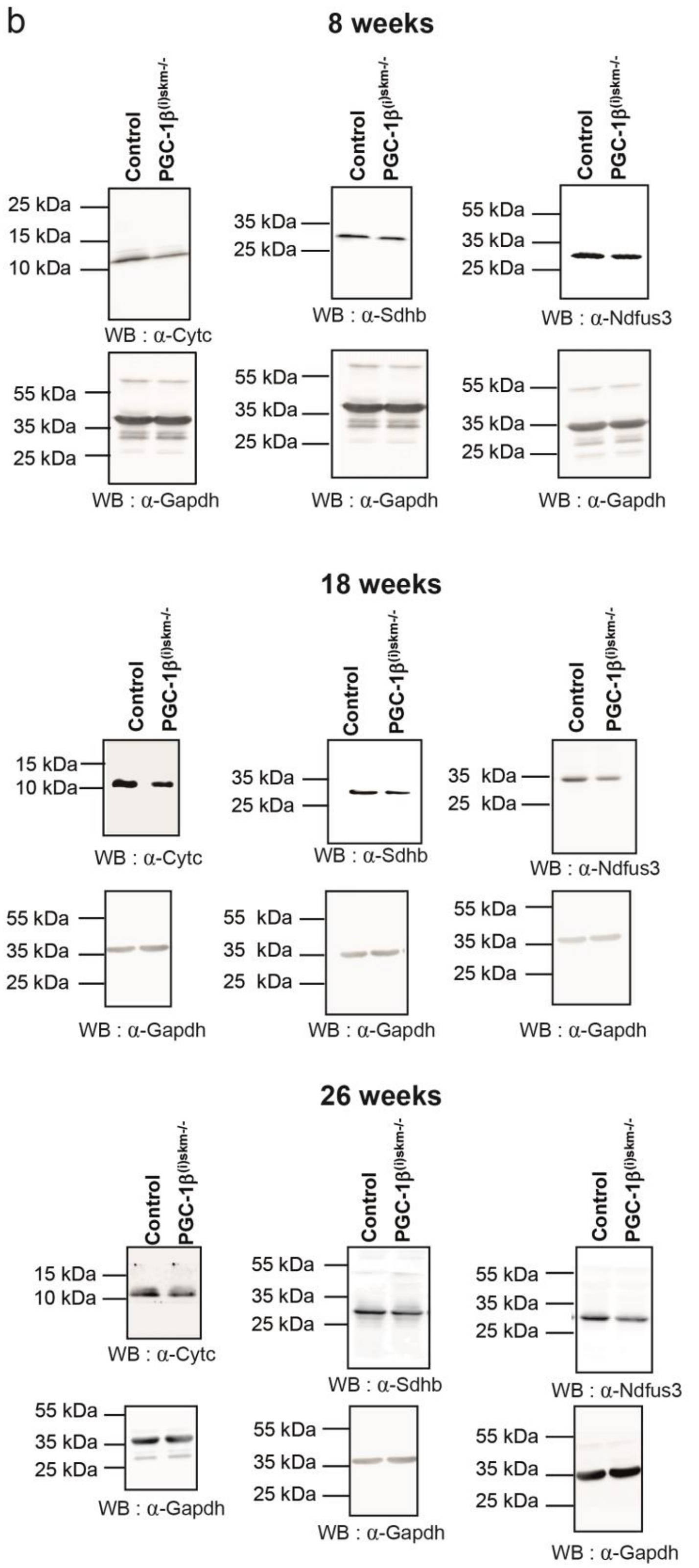

b
8 weeks
Control
PGC-1β(i)skm-/-
25 kDa
15 kDa
10 kDa
WB : α-Cytc
35 kDa
25 kDa
WB : α-Sdhb
55 kDa
35 kDa
25 kDa
WB : α-Ndfus3
WB : α-Gapdh
18 weeks
WB : α-Cytc
WB : α-Sdhb
WB : α-Ndfus3
WB : α-Gapdh
26 weeks
WB : α-Cytc
WB : α-Sdhb
WB : α-Ndfus3
WB : α-Gapdh

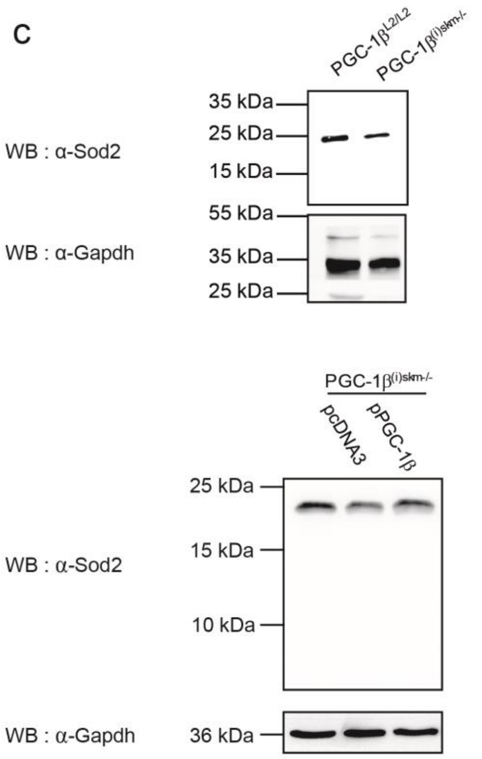

Supplementary Figure 10. Uncropped scans of western blots shown in Figure 1d **(a)**, Figure 5e **(b)**, and Figure 7f and k **(c)**. Data presented in Figure 1d are boxed in panel **(a)**.

Supplementary Table 1

List of primers used for PCR or RT-qPCR analyses of the indicated genes.

Gene	Primer (5' – 3')	Gene	Primer (5' – 3')
**PGC-1β**	1:CCTGTCTCTGCTTCCTAAGTATTGTGC<br>2:GTGAGGTTGGATCTGCTTATCCACTG<br>3:CTCTGGGGCCTCATGAGCTAATG	**Nd1**	CCCATTCGCGTTATTCTT<br>AAGTTGATCGTAACGGAAGC
**GAPDH (DNA IC)**	GCAGCTGCTCAGCTGCCTGC<br>GATCGCACTTCTCATACTCG	**Ndufs3**	CTGACTTGACGGCAGTGGAT<br>CATACCAATTGGCCGCGATG
**Cre**	TTCCCGCAGAACCTGAAGATGTTCG<br>GGGTGTTATAAGCAATCCCCAGAAATGC	**Ndufv1**	ATCGCTCGACAGACATTGTG<br>GTGGCCTTCTATCTGCTTGC
**Hprt**	GTTGGATACAGGCCAGACTTTGTTG<br>GATTCAACTTGCGCTCATCTTAGGC	**Ndufab1**	CACCCCCACTGACGTTAGAC<br>TCGTCTTCCATGGCCATAAT
**PGC-1β**	TGCGGAGACACAGATGAAGA<br>GGCTTGTATGGAGGTGTGGT	**Ndufb3**	AAGGGACGCCATTAGAAACG<br>TACCACAAACGCAGCAAACC
**PGC-1α**	AAGTGTGGAACTCTCTGGAACTG<br>GGGTTATCTTGGTTGGCTTTATG	**Ndufb7**	GGCCACACAACAAGAGATGA<br>GCGTTTCACGTAATCCAGGT
**Mhc I**	AGATGAATGCCGAGCTCACT<br>CTCATCCAAACCAGCCATCT	**Sdhb**	TGGTGGAACGGAGACAAGTA<br>TGGCAGCGGTAGACAGAGAA
**Mhc IIA**	AATCGAGGCCCAGAATAGGC<br>TCTTCTTTCACGGTCAGGGT	**Sdhd**	GATCCCTGCTGGGTACTTGA<br>AAGTAGCAAAGCCCAGCAAA
**Mhc IIX**	CAAGACCGAAGGCGGAACTA<br>TGACAGTGACGCAGAACAGG	**Cytc**	AGAAGGGAGAAAGGGCAGAC<br>TGATCTGAATTTGGTGTGTGAA
**Mhc IIB**	ACGCTTGCACACAGAGTCAG<br>TCACAGTCATGGCGAGCTG	**Uqcrc1**	GGGGCAAAAACATCCTTAGG<br>ATCCGGCTCTCCCACTCAGC
**Acadm**	GGATGACGGAGCAGCCAATG<br>ATACTCGTCACCCTTCTTCT	**Uqcrfs1**	TGGTCTCCCAGTTTGTTTCC<br>GCAGCTTCCTGGTCAATCTC
**Acadvl**	TATCTCTGCCCAGCGACTTT<br>TGGGTATGGGAACACCTGAT	**Uqcr11**	TGCTGAGCAGGTTTCTAGGC<br>TCCTTCTTAAACTTGCCGTTG
**Dlat**	TCCTGCAGGTGTCTTCACAG<br>GACGGAGATTTTCCCTTTCC	**Cox2**	AATTAGCTCCTTAGTCCTCT<br>CTTGGTCGGTTTGATGTTAC
**Fh1**	AGCAATGCATATTGCTGCTG<br>CGCATACTGGACTTGCTGAA	**Cox4i**	TTCAGTTGTACCGCATCCAG<br>GGATGGGGCCATACACATAG
**Mdh1**	GAAGCCCTGAAAGACGACAG<br>TCGACACGAACTCTCCCTCT	**Cox5b**	CGTCCATCAGCAACAAGAGA<br>AGATAACACAGGGGCTCAGT
**Pdhb**	TCGAAGCCATAGAAGCCAGT<br>AGGCATAGGGACATCAGCAC	**Atp5l**	CCCCTGCTGAAATCCCTACA<br>TAAAACCACATCCACACCTC
**Idh3b**	ATCTGAGCGAGGTGCAGAAT<br>TACGTTGGCAAACAAATCCA	**Atp50**	GCAACACCCAGGGTATCATC<br>TTGGTTTGGACTCAGGAAGC
**Errγ**	CAAGCGGACATCCTCGGG<br>GTAGCTAAGGTCCCTCGTGC	**Fas**	ACCCAAGCATCATTTTCGTC<br>AGGATATGGAGAGGGCTGGT
**Opa1**	GATGACACGCTCTCCAGTGA<br>TCGGGGCTAACAGTACAACC	**Mfn1**	CCTCCATGGGCATCATCGTT<br>TGCAGCTTCTCGGTTGCATA
**Mfn2**	CTCAGGAGCAGCGGGTTTAT<br>GAGAGGCGCCTGATCTCTTC	**Mrpl55**	AGGACGGCTCCACTATCCAT<br>ACTGTCCACTACCTCTGGCT
**Drp1**	AGAAAACTGTCTGCCCGAGA<br>GCTGCCCTACCAGTTCACTC	**Mrpl47**	AAGCGACAGAGGTTGCCAAT<br>CCACTGCTTGAATTTGTGCCA
**Fis1**	CCGGCTCAAGGAATATGAAA<br>ACAGCCAGTCCAATGAGTCC	**Mrps35**	ATGGACGAGTATGTGTGGGC<br>TCGTTCTCCCCCTCGTTCTT
**Tomm40l**	GAAGAGGGGGCCATCTTGAC<br>GGGCAGAGTCAGGTGGTAAC	**Sod1**	CCAGTGCAGGACCTCATTTT<br>TTGTTTCTCATGGACCACCA
**Timm44**	GGCCTTAACCGACAAGGTCA<br>GTCAAGCTCCCCGGAAATCA	**Sod2**	CCGAGGAGAAGTACCACGAG<br>GCTTGATAGCCTCCAGCAAC
**Timm8a1**	TGCACCAGATGACGGAACTT<br>CGGGTTTGGACTTCTGGGTC	**Sod3**	TCTGCAGGGTACAACCATCA<br>ACCTCCATCGGGTTGTAGTG
**Mtg1**	GTCGGTGTCCCCAATGTAGG<br>TTTCAATCCGAGGAGCCAGC	**Gpx1**	GTCCACCGTGTATGCCTTCT<br>TCTGCAGATCGTTCATCTCG

**Tsfm**	AGTTGGTCCAGCAAGTAGCC<br>CAATCGCTAGGGCCAACTGA	**Nrf-1**	GCACCTTTGGAGAATGTGGT<br>CTGAGCCTGGGTCATTTTGT
**Gfm1**	GGCAGGTTTACACGGGAAGA<br>TGGGGCAACAATGGTCTCTC	**Gabpa**	TTCACCGGGGAACAGAACAG<br>ACGTTGTCCCCATTTTTGCG
**Mrpl3**	AGATGAGCCGTGGCCTTTAC<br>GTCAGGGCTGCGATTTTTCC	**Tfam**	AATTGCAGCCATGTGGAGGGA<br>GCTCTCAGGTGGGATGCAG
**Mrpl18**	TGTGGCCTAACCGTGAGTTC<br>CACAAGCCACCACGTTTCTG	**Errα**	GCAAAGTCCTGGCCCATTTC<br>GGCTAACACCCTATGCTGGG
**Nd2**	GGGCATGAGGAGGACTTAACCAAAC<br>TGAGGTTGAGTAGAGTGAGGGATGG	**Errβ**	GGGAGCTTGTGTTCCTCATC<br>ATCTCCATCCAGGCACTCTG
**Cox1**	GCCTTTCAGGAATACCACGA<br>AGGTTGGTTCCTCGAATGTG	**Nd4**	CCACTGCTAATTGCCCTCAT<br>CTTCAACATGGGCTTTTGGT
**CytB**	ATTCCTTCATGTCGGACGAG<br>ACTGAGAAGCCCCCTCAAAT	**Cox3**	CAAGGCCACCACACTCCTAT<br>ATTCCTGTTGGAGGTCAGCA
**Tfb1**	GGAAGCAAACAGCACAGTCG<br>GCTGCTTGATCTTGGGCTCT	**Atp6**	CCTTCCACAAGGAACTCCAA<br>GGTAGCTGTTGGTGGGCTAA
**Pgs**	ACACAGGTTCCAGTGGATCAG<br>TTTATCTGCCCCTTCATGAGC	**Tfb2**	CCCGTGGACATCCAGGAATC<br>CCACTCTGGCACCAGCTTTA
**Taz**	GACCCTCATCTCTGGGGGAT<br>CAGCTCCTTGGTGAAGCAGA	**Ptpmt**	GCAACACCTCGAAGGAATGG<br>GAGATTGGCCAAGGTTGGGA
**Lclat1**	GTTGTGACCCCCGTGGAG<br>TCCATGACACCATGATTCTGAC		
